# Ultrasonication‐Induced Iridium Single‐Atom Decoration on Anodized NiO Nanosponge Electrocatalyst for Enhanced Oxygen Evolution Reaction

**DOI:** 10.1002/smll.75110

**Published:** 2026-08-17

**Authors:** Nastaran Farahbakhsh, Mohammad Hossein Enayati, Mahmoud Monirvaghefi, Seyedeh Zahra Sajadian, Jean Marie Vianney Nsanzimana, Majid Shahsanaei, Manuela S. Killian

**Affiliations:** ^1^ Chemistry and Structure of Novel Materials Department of Chemistry and Biology University of Siegen Siegen Germany; ^2^ Department of Materials Engineering Isfahan University of Technology Isfahan Iran; ^3^ Department of Industrial Engineering University of Padova Padova Italy

**Keywords:** chemistry, cyclic voltammetry, electrocatalyst, electrochemistry, iridium, materials science, nickel oxide, oxygen evolution, scanning transmission electron microscopy, x‐ray photoelectron spectroscopy

## Abstract

This study develops a facile strategy to engineer active and stable Ir single‐atom sites on nanostructured NiO to enhance the oxygen evolution reaction (OER). Electrochemical anodization is used to synthesize a NiO nanosponge, and sonochemical treatment is employed to simultaneously generate surface defects and decorate NiO with Ir single atoms. This method introduces active anchoring points and a favorable electronic environment to improve Ir stabilization and enhance electrocatalytic activity. Systematic characterizations, including high‐resolution transmission electron microscopy (HR‐TEM) and aberration‐corrected HAADF‐STEM, confirm the atomic dispersion of Ir for NiO decorated with 0.5 mM Ir (NiO‐0.5 Ir), and X‐ray photoelectron spectroscopy (XPS) demonstrates the formation of Ir^3+^ and Ir^4+^ chemical states. Time‐of‐flight secondary ion mass spectrometry (ToF‐SIMS) and principal component analysis (PCA) confirm the highest NiIr^−^ fragment intensity and higher Ir‐Ni interaction values (NiIr^−^/Ir^−^) for the NiO‐0.5 Ir electrode. Electrochemical measurements demonstrate enhanced OER activity for NiO‐0.5 Ir, exhibiting an overpotential of 290 mV at 10 mA cm^−2^ and a Tafel slope of 78 mV dec^−1^, delivering 40 mA cm^−2^ at 1.6 V vs. RHE in 1.0 M KOH, which is twice the performance of IrO_2_/Ni. This approach facilitates the development of durable and highly active single‐atom catalysts for high‐performance OER.

## Graphical abstract

A facile one‐pot sonochemical strategy generates Ir single‐atom catalysts on nanostructured NiO for enhanced oxygen evolution reaction performance. Surface defects enable strong anchoring of Ir single atoms on NiO. The NiO‐0.5 Ir catalyst exhibits significantly improved activity, supported by structural, spectroscopic, and electrochemical analyses indicating strong Ir‐Ni interactions and accelerated reaction kinetics.

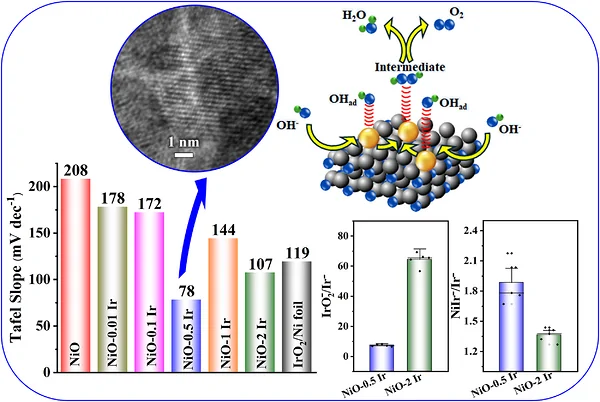

## 1 Introduction

The oxygen evolution reaction (OER) is essential for advancing sustainable development and enhancing energy conversion technologies. Due to the inherently slow rate of OER, which involves four proton‐coupled electron transfers (2H_2_O → O_2_ + 4H^+^ + 4e^−^), effective OER catalysts are critical for speeding up the reaction and lowering the overpotential [1]. Iridium oxide (IrO_2_) and Ruthenium oxide (RuO_2_)‐based catalysts are commonly employed to improve the sluggish kinetics of OER due to their excellent durability and catalytic performance [2]. However, the large‐scale application of these noble metals has been significantly limited due to their high cost and limited availability. To fully utilize noble metal atoms and their distinct active sites, the catalyst should be precisely engineered at the single‐atom scale [3, 4].

Single‐atom catalysts (SACs) have attracted widespread attention as highly efficient catalysts because of their unique properties, such as maximized atomic utilization, abundant active sites, specific metal coordination, improved interaction with reactant molecules, and cost‐effectiveness [5, 6, 7, 8, 9, 10]. However, the high surface energy of single atoms causes them to aggregate into nanoclusters or nanoparticles, diminishing their unique catalytic characteristics. Thus, one of the critical challenges in SACs research is developing reliable synthetic methods that enable stable anchoring and uniform distribution of single atoms on suitable supports to ensure optimal catalytic efficiency and durability [11]. Several techniques have been explored for synthesizing SACs, including atomic layer deposition (ALD), galvanic replacement, vapor deposition, impregnation, and electro/photo deposition [12, 13, 14, 15]. Among these techniques, engineering atomic‐scale defects to anchor single atoms is a promising approach with significant potential for achieving stable and controllable decoration [16, 17].

Notably, incorporating SACs can significantly boost the intrinsic OER activity of previously developed catalysts, such as carbon‐based materials, metals, and metal oxides [18, 19, 20]. Since the interaction between SACs and their support material is critical in determining the catalyst's intrinsic OER activity and stability, selecting appropriate support is essential for achieving high OER performance [13, 21]. Nickel oxide (NiO) is an effective electrocatalyst for OER in alkaline environments, primarily due to its favorable adsorption‐desorption energy balance with oxygen‐based intermediates [22]. For instance, Liang et al. prepared a NiO/Ni composite material and investigated its electrochemical performance, demonstrating notable OER activity with an overpotential of 345 mV at a current density of 10 mA cm^−^^2^ in a 1.0 M KOH solution [23]. In contrast to carbon‐based materials that tend to oxidize under anodic conditions, NiO undergoes a surface transformation into nickel oxyhydroxide (NiOOH) under oxidative potentials, forming a few atomic layers. NiOOH is widely considered the key active phase in driving OER [24, 25]. As highlighted by Markovic et al., NiOOH exhibits an optimal binding strength with hydroxyl intermediates (OH_ad_) compared to other 3d transition metals, such as Co, Fe, and Mn, thus providing theoretical insight for designing efficient alkaline OER catalysts [25]. Moreover, tailoring the Ni‐based materials into nanostructured forms can significantly enhance their intrinsic electrocatalytic performance [26, 27]. Research indicates that the anodic fabrication of nanosponge NiO provides multiple benefits, such as an increased surface area, binder‐free immobilization, enhanced structural stability, and efficient electron transfer to the underlying Ni substrate facilitated by its porous architecture [26, 28].

Therefore, combining nanostructured NiO with Ir SA could enhance the reaction kinetics and improve OER performance. However, the actual effectiveness and practicality of using NiO to improve both the reactivity and stability of Ir require further validation.

Inspired by these advantages, we developed a facile approach to synthesize a single‐atom Ir catalyst (termed NiO‐x Ir), featuring ultrahigh dispersion of Ir atoms on nanostructured, self‐supported anodically grown NiO. This strategy utilizes a one‐pot synthesis method that simultaneously introduces surface defects and anchors Ir atoms onto these defect‐rich sites. The generated defects within the nanostructured NiO serve as electrochemically active sites, enabling strong and stable immobilization of Ir single atoms (SAs) as efficient co‐catalysts. A main innovation of this approach lies in the precise control of NiO surface chemistry through tunable sonochemical treatment, leading to both tailored surface modification and uniform Ir SA decoration in a single step. As a result, the well‐dispersed Ir SAs significantly enhanced catalytic performance, delivering up to a 4.4‐fold improvement in oxygen evolution reaction (OER) activity compared to pristine NiO.

## 2 Experimental Section

### 2.1 Materials

Nickel foil (0.125 mm thickness, 99.9%, ChemPur), potassium hydroxide (KOH, 85%, Sigma–Aldrich), ethylene glycol (C_2_H_6_O_2_, 99.8%, Carl Roth), hydrochloric acid (HCl, VWR Chemicals), ammonium fluoride (NH_4_F, Sigma–Aldrich), orthophosphoric acid (H_3_PO_4_, 85%, VWR Chemicals), dihydrogen hexachloroiridate(IV) hydrate (H_2_IrCl_6_·xH_2_O, ≥99%, Alfa Aesar), and iridium(IV) oxide (IrO_2_, 99.99%, Thermo Scientific) were used as received without further purification.

### 2.2 Fabrication of NiO Nanostructures

Nickel foil (99.9% purity, 0.125 mm thick) was cut into 1.5 × 1.5 cm^2^ squares, ultrasonically cleaned in ethanol and deionized water (5 min each), and dried under nitrogen. Anodization was conducted in two steps: first, electrochemical polishing in a solution of 0.5 M HCl and 2 vol% deionized water in ethylene glycol at 6 V and 20°C for 15 min to remove surface oxides. The samples were then rinsed with deionized water and isopropanol. In the second step, nanostructured, self‐supporting NiO layers were formed by anodizing in 0.5 M NH_4_F in 85% H_3_PO_4_ at 6 V and 20°C for 3 h. Post‐anodization, the electrodes were rinsed, soaked in ethanol for 10 min, and dried under nitrogen. The process used a two‐electrode setup powered by a Keithley 2000 source meter, with Ni as the working electrode and Pt as the counter electrode. Finally, the anodized electrodes were annealed in air at 450°C for 12 h.

### 2.3 Ir SAs Decoration

NiO decorated with Ir single atoms (SAs) was synthesized using a sonochemical method with a Hielscher probe sonicator operating at 40% amplitude, 1500 W power density, and a duty cycle of 0.5. The aqueous solution for Ir SA deposition contained varying concentrations of chloroiridic acid (H_2_IrCl_6_·xH_2_O, 99.9%, Alfa Aesar): 0.01, 0.1, 0.5, 1, and 2 mM Ir, in a total volume of 30 mL of deionized water. Before sonication, the NiO electrode immersed in the solution was purged with argon for 10 min to eliminate dissolved oxygen. Defect generation and Ir SA decoration were carried out simultaneously through sonication for 2 h under continuous argon flow, with the vessel placed in a water‐ice bath to regulate temperature and prevent overheating. After treatment, the NiO‐x Ir electrodes were thoroughly rinsed with deionized water and dried in an oven at 37°C for 24 h.

### 2.4 Fabrication of IrO_2_ Reference Electrode

The IrO_2_/Ni foil electrode was produced using an ink solution containing iridium oxide (IrO_2_, 99.9%, Thermo Scientific) at a density of 10 mg/cm^2^ in a mixture of 500 µL ethanol and 20 µL Nafion. The ink solution was ultrasonicated for 30 min, and 130 µL of the ink was drop‐cast onto the nickel foil, followed by drying in a vacuum oven at 60°C. This step was repeated four times.

### 2.5 Characterization of Electrodes

The surface morphology of the NiO and NiO‐x Ir samples was characterized using a field emission scanning electron microscope (FE‐SEM, Zeiss 55) equipped with an energy‐dispersive X‐ray spectroscopy (EDS) system. X‐ray photoelectron spectroscopy (XPS) was conducted using a Surface Science Instruments S‐probe spectrometer with an Al Kα radiation source to investigate the elemental composition and oxidation states of the electrodes. Data analysis was performed with the CasaXPS software suite, applying calibration based on the C 1s peak positioned at 284.8 eV. Time‐of‐flight secondary ion mass spectrometry (ToF‐SIMS) measurements were performed using a ToF‐SIMS 4 system (IONTOF, Germany) equipped with a 25 keV Bi^+^ liquid metal ion source. The data were analyzed using SurfaceLab 7.3 software. Principal component analysis (PCA) of the spectra was performed using Spectragui NESAC/BIO. Before analysis, the data were normalized to the total intensity of all selected peaks and square‐root mean‐centered. Crystallographic analysis of the NiO‐Ir electrodes was conducted using X‐ray diffraction (XRD) with a Panalytical X'Pert Pro diffractometer that employed Cu Kα radiation. Transmission electron microscopy (TEM) imaging was performed on a Thermo Fisher Scientific FEI Talos microscope operating at 200 kV, equipped with a SuperX EDS detector for elemental analysis. Further compositional and structural investigations were performed using a Thermo Fisher Titan microscope (TEAM 1), equipped with aberration correction for probe and image modes and operated at 300 kV. High‐resolution transmission electron microscopy (HR‐TEM) and elemental mapping were obtained using a FEI TITAN G2 60–300 microscope. Aberration‐corrected high‐angle annular dark‐field scanning transmission electron microscopy (HAADF‐STEM) images were collected using a double aberration‐corrected Spectra 300 (Thermo Fisher Scientific) microscope.

### 2.6 Electrochemical Measurements

Electrochemical measurements were conducted using a three‐electrode setup connected to a BioLogic SP‐200 instrument. The working electrodes included nanostructured NiO and Ir single‐atom‐decorated NiO, each measuring 1 cm × 1 cm. These electrodes were used as prepared, without any additional modifications. A platinum foil served as the counter electrode, while an Hg/HgO electrode in 1.0 M KOH was used as the reference electrode. All potentials recorded relative to the Hg/HgO electrode were converted to values against the reversible hydrogen electrode (RHE) using the equation: E_RHE_ = E_Hg/HgO_ + 0.059 × pH + 0.098 V. The overpotentials (η) were calculated using the equation η = E_RHE_ –1.23 V.

Before OER measurements, the electrode surface was activated by performing 20 cycles of cyclic voltammetry (CV) in the potential range of 0.85–1.55 V vs. RHE at a scan rate of 100 mV·s^−1^. Following activation, the catalytic performance was evaluated using linear sweep voltammetry (LSV) at a scan rate of 5 mV·s^−1^. All polarization curves were corrected for ohmic losses using the equation: E = E_RHE_ – iR_s_, where the solution resistance (R_s_) was obtained from EIS Nyquist plots. Tafel slopes were extracted from the oxygen evolution reaction (OER) polarization curves recorded under the same scan conditions. Electrochemical impedance spectroscopy (EIS) was carried out using a 10 mV AC amplitude over a frequency range of 100 kHz to 100 mHz. Mott–Schottky analysis was performed using impedance spectroscopy with a 5 mV root‐mean‐square (RMS) AC signal over a frequency range of 200 kHz to 1 Hz. The applied potential was swept from −0.5 to 0.6 V vs. Hg/HgO. Mott‐Schottky plots were generated from impedance data collected at 15 kHz. To estimate the double‐layer capacitance (C_dl_), cyclic voltammetry (CV) was performed within a non‐Faradaic potential window at various scan rates (2, 5, 10, 15, 25 mV s^−1^). The C_dl_ was calculated by plotting half the difference between anodic and cathodic current densities (Δj/2) against the scan rate. The ECSA was calculated by dividing C_dl_ by the specific capacitance (C_s_) of NiO (40 µF cm^−2^). The stability of the catalysts was assessed through 1000 CV cycles at 100 mV s^−1^ and a 24‐h chronoamperometry test at current densities of 10 and 20 mA cm^−2^. The turnover frequency (TOF) was calculated via the following equation [29, 30]:

(1) $$TOF=\frac{I}{4nF}$$

where the term 4 corresponds to the number of electrons involved in the OER, F represents the Faraday constant (96,485 C mol^−1^), and n stands for the number of active sites, calculated using the following equation [29, 31]:

(2) $$n=\frac{Q}{F\;A}$$

Here, A represents the testing area of the electrode, and Q denotes the total charge of the redox peaks, which can be determined by integrating the reductive peak in the redox range area of the backward cyclic voltammetry (CV) scan in the active region [29, 30].

## 3 Results and Discussion

### 3.1 Synthesis and Optimization of the Catalyst

The performance of sonochemically Ir‐decorated nanostructured NiO is investigated for the electrocatalytic oxygen evolution reaction (OER). The NiO nanosponge was synthesized via a two‐step anodization process followed by annealing in air to obtain crystalline NiO, based on the procedure reported by Sultan et al. [28]. The anodization time was varied (1, 3, 6, and 16 h) to study its effect on OER performance. FE‐SEM images (Figure S1) and ion‐milled cross‐sections (inset of Figure S1) show that the nanostructure is homogeneous across the entire surface, regardless of anodization time. However, increasing the anodization duration led to a thicker anodic layer. Electrochemical measurements revealed that the sample anodized for 3 h exhibited the best OER performance, with the lowest overpotential, Tafel slope, and charge transfer resistance (Figure S2 and Table S1). Further discussion is provided in Note S1. As a result, the 3 h anodized sample was selected for subsequent modification and is referred to hereafter as pristine nanostructured NiO (NiO).

Iridium precursors at various concentrations were subsequently deposited onto the nanostructured NiO using a sonochemical method to produce Ir‐decorated NiO (NiO‐x Ir) electrodes, which were then evaluated for electrocatalytic oxygen evolution. The schematic of the synthesis process is shown in Figure 1. The NiO layer had a thickness of approximately 3 µm, as confirmed by TEM images of FIB‐cut nanostructured NiO (Figure S3a).

**FIGURE 1:**
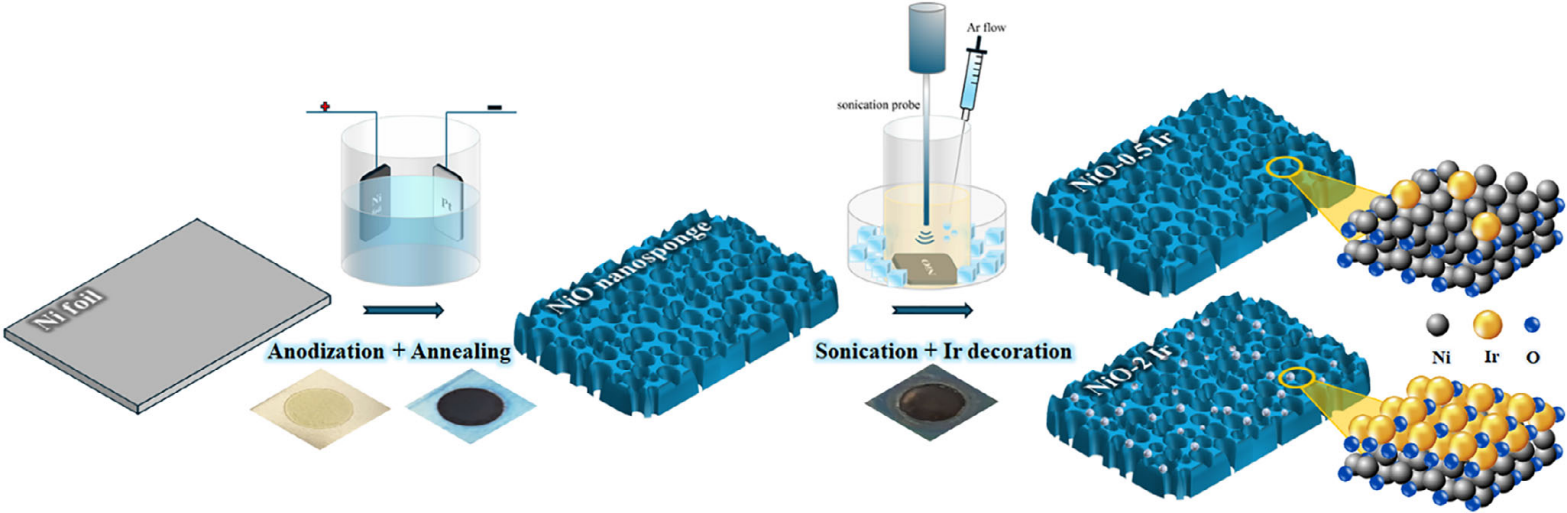
Schematic illustration of the synthetic process for in situ anodic growth of nanostructured NiO, followed by surface modification using a sonochemical method to decorate NiO with Ir, forming NiO‐x Ir.

### 3.2 Morphological and Microstructural Characterization

Field‐emission scanning electron microscopy (FE‐SEM) was used to examine the morphology of the NiO nanosponge catalyst. As shown in Figure 2a, the structure exhibits a porous morphology with pore sizes ranging from 10 to 20 nm. Figure S3b reveals that the porous structure extends to the cracked edges, forming a homogeneous porous network. FE‐SEM images after Ir decoration at different precursor concentrations are shown in Figure 2b (0.5 mM Ir, NiO‐0.5 Ir), Figure 2c (2 mM Ir, NiO‐2 Ir), and Figure S3c–e (NiO‐0.01 Ir, NiO‐0.1 Ir, NiO‐1 Ir). Interestingly, all samples displayed similar nanostructures, indicating that the incorporation of Ir does not alter the overall morphology of the NiO nanosponge. However, IrO_X_ nanoparticles were observed for the NiO‐2 Ir electrode (highlighted with red circles in Figure 2c) and NiO‐1 Ir electrode (Figure S3e), whereas no such particles were detected on the NiO‐0.5 Ir electrode (Figure 2b) or at lower concentrations.

**FIGURE 2:**
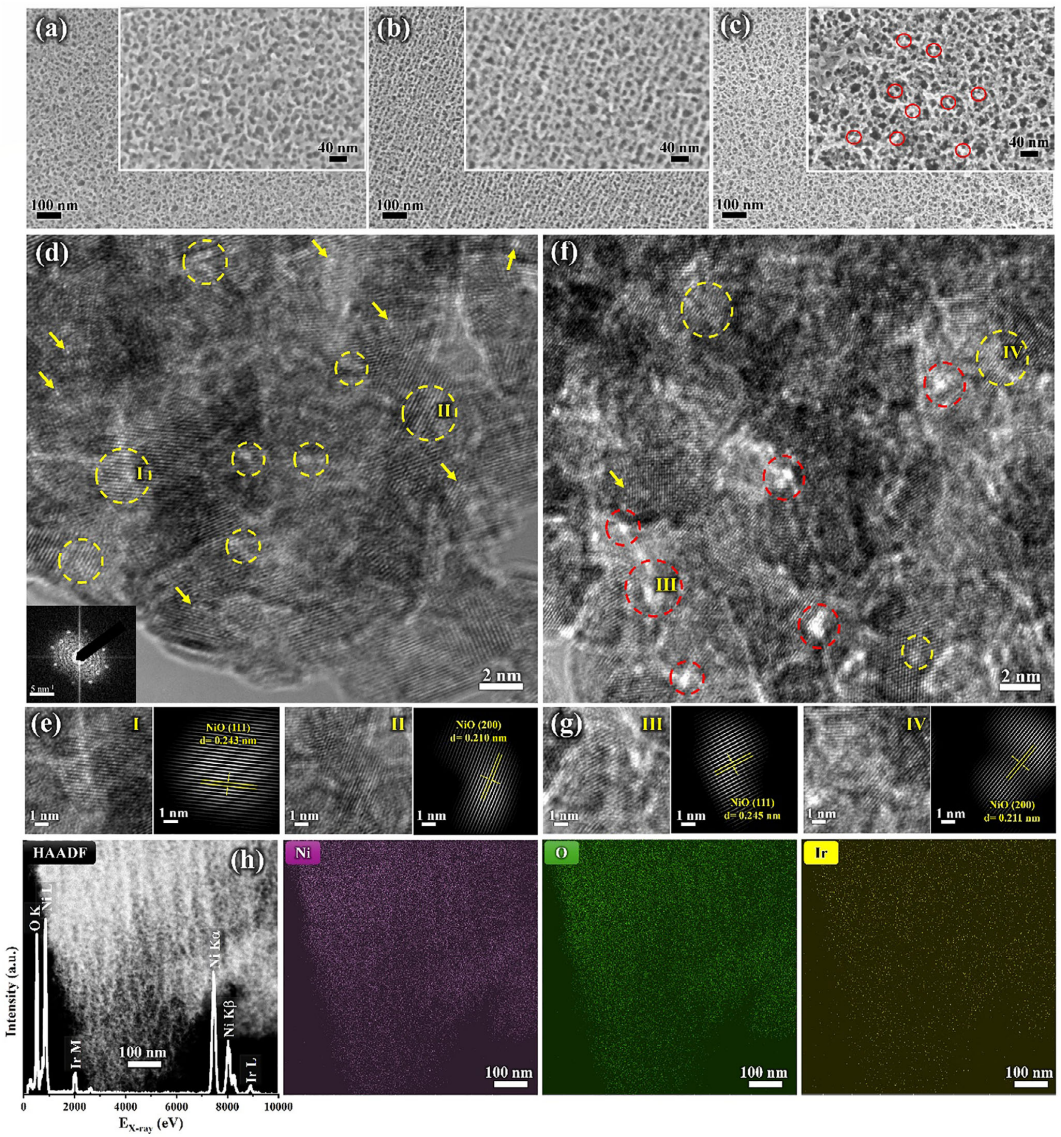
FE‐SEM images of (a) NiO, (b) NiO‐0.5 Ir, and (c) NiO‐2 Ir (insets show higher magnification); (d) HR‐TEM image of NiO‐0.5 Ir (inset shows the SAED pattern of NiO); (e) Inverse FFT image of NiO lattice fringes from regions I and II in Figure 2d; (f) HR‐TEM image of NiO‐2 Ir; (g) Inverse FFT image of NiO lattice fringes from regions III and IV in Figure 2f; (h) HAADF‐STEM image of the NiO‐0.5 Ir catalyst with inset showing the EDS spectrum and accompanying EDS elemental mappings.

To better understand the structural effects of Ir decoration via sonochemical treatment, high‐resolution transmission electron microscopy (HR‐TEM) was performed on NiO nanostructures decorated with 0.5 and 2 mM Ir precursors, as shown in Figure 2d,f, respectively. Ir atoms, being significantly heavier than Ni or O, exhibit a much higher scattering ability, which results in bright and distinct spots in the HR‐TEM images. Figure 2d clearly shows well‐defined, isolated bright spots (highlighted with yellow circles) on the NiO‐0.5 Ir electrode, which contrast sharply with the lighter atoms of the NiO support. This indicates the presence of dispersed Ir atoms at the atomic scale. The selected area electron diffraction (SAED) pattern, shown in the inset of Figure 2d, provides crystallographic information for the NiO‐0.5 Ir sample. The inverse fast Fourier transform (FFT) analysis from regions I and II in Figure 2d, as presented in Figure 2e, reveals lattice fringes of approximately 0.243 and 0.210 nm, which correspond to the (111) and (200) planes of face‐centered cubic (FCC) NiO, respectively [32].

In the NiO‐2 Ir electrode, Figure 2f illustrates the presence of IrO_x_ nanoparticles distributed across the NiO surface (highlighted in red), as well as isolated Ir atoms and small clusters (highlighted in yellow). The FFT analyses of regions III and IV in Figure 2f, shown in Figure 2g, display lattice spacings of about 0.245 and 0.211 nm, which are consistent with the (111) and (200) crystallographic planes of cubic NiO, respectively [33]. Although a higher concentration of Ir precursor was used, no distinguishable lattice fringes corresponding to Ir or IrO_x_ phases were observed. This implies that the Ir species are either highly dispersed at the atomic level or present as amorphous nanoparticles, making them undetectable in the HR‐TEM lattice images. Furthermore, the HAADF‐STEM image in Figure 2h, along with the corresponding EDS elemental mapping for NiO‐0.5 Ir, which did not show larger particles, confirms the presence of Ir on NiO. The EDS spectrum shown in the inset of Figure 2h further corroborates the existence of Ir species on NiO in the NiO‐0.5 Ir electrode.

Aberration‐corrected high‐angle annular dark‐field scanning transmission electron microscopy (HAADF‐STEM) images (Figure 3a) show isolated bright spots corresponding to Ir atoms, arising from Z‐contrast relative to the NiO support in the NiO‐0.5 Ir electrode, thereby confirming the atomic dispersion of Ir species [34, 35, 36]. Similar features are also observed in additional regions (Figure S4a,b). In contrast, aberration‐corrected HAADF‐STEM images of NiO‐2 Ir (Figure 3c and Figure S4c,d) reveal the formation of IrO_x_ nanoclusters together with isolated Ir atoms, suggesting that higher Ir loading leads to partial aggregation of Ir species. Intensity profiles from three line regions of NiO‐0.5 Ir (Figure 3b) show clear intensity peaks corresponding to individual Ir atoms, confirming their isolated distribution on the NiO surface. For NiO‐2 Ir (Figure 3d), line 1 indicates isolated Ir atoms, whereas line 2 shows multiple adjacent sharp peaks and line 3 exhibits broadened intensity features, both consistent with the formation of IrO_x_ nanoclusters [36].

**FIGURE 3:**
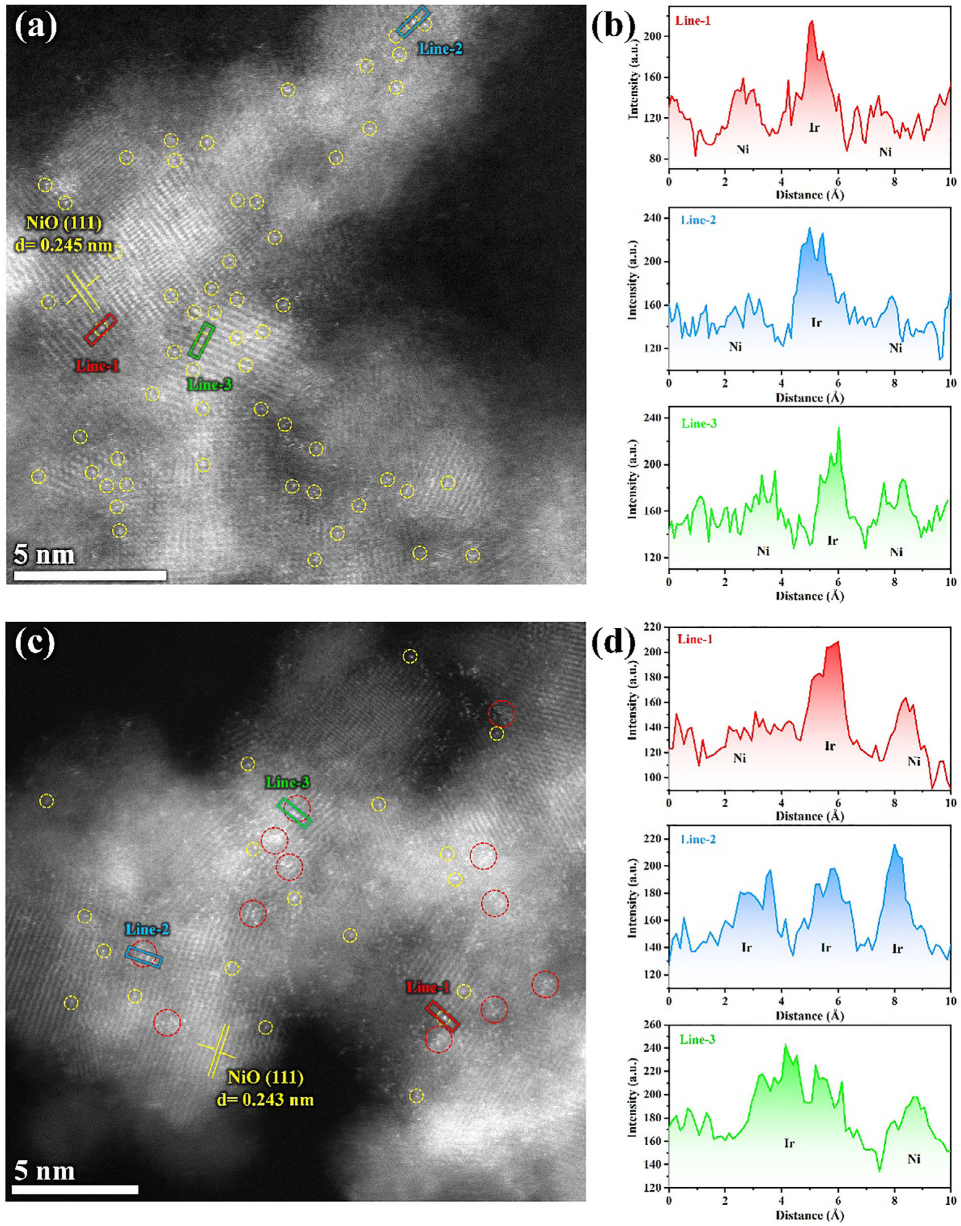
(a) Aberration‐corrected HAADF‐STEM image of NiO‐0.5 Ir, with yellow circles indicating Ir single atoms, and (b) corresponding line‐scan intensity profiles recorded along lines 1–3 in (a); (c) aberration‐corrected HAADF‐STEM image of NiO‐2 Ir, with yellow circles marking Ir single atoms and red circles indicating IrO_x_ nanoclusters, and (d) corresponding line‐scan intensity profiles recorded along lines 1–3 in (c).

### 3.3 Structural and Chemical Characterization

Figure 4a shows the XRD patterns of nanostructured NiO before and after adding Ir at different concentrations. All samples exhibit broad diffraction peaks, characteristic of materials with a nanostructured oxide layer. The peaks at 37.3°, 43.3°, and 62.9° correspond to the (111), (200), and (220) crystal planes of face‐centered cubic NiO, matching the standard JCPDS 47–1049 data [37]. Additionally, peaks related to metallic Ni are observed in all samples at 44.5°, 51.8°, and 76.4°, corresponding to the (111), (200), and (220) planes of Ni, respectively, according to JCPDS 04–0850.

**FIGURE 4:**
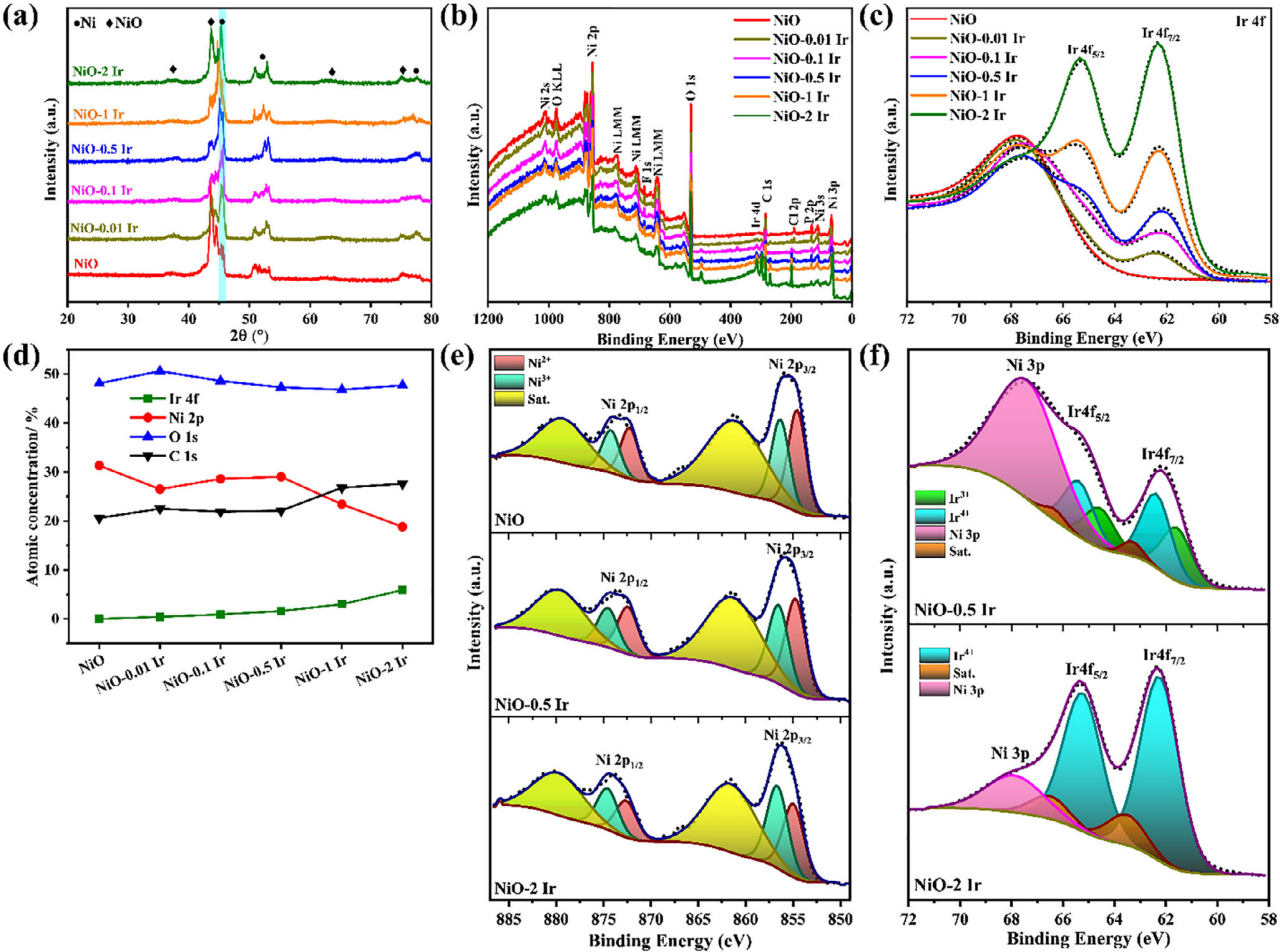
(a) XRD pattern of pristine NiO and NiO‐x Ir electrodes; (b–d) Overall XPS spectra of pristine NiO and NiO‐x Ir electrodes: (b) survey spectrum; (c) Ir 4f; (d) atomic percentages of the elements detected by XPS on the surface of NiO‐x Ir electrodes; (e) deconvolution of Ni 2p spectra of pristine NiO, NiO‐0.5 Ir and NiO‐2 Ir; (f) deconvolution of Ir 4f spectra of NiO‐0.5 Ir and NiO‐2 Ir.

No clear peaks for Ir or Ir oxides appear in the Ir‐modified samples, likely because the Ir content is too low for XRD detection. However, after depositing Ir on NiO using the sonochemical method, the NiO peaks become weaker and broader, indicating increased disorder in the crystal structure. This change may result from interactions between Ir atoms and surface defects in the NiO lattice. At the same time, the metallic Ni peaks become sharper, especially in the NiO‐0.5 Ir sample, which shows the strongest Ni peak. Additionally, in the NiO‐0.5 Ir and NiO‐1 Ir samples, the Ni peak shifts to lower angles, with the most significant shift observed in NiO‐1 Ir. The incorporation of Ir may cause lattice expansion, leading to the peak shift. When the Ir concentration increases to 2 mM (NiO‐2 Ir), the peaks of both Ni and NiO become sharper, with no observable shift. This suggests reduced lattice distortion in NiO, possibly due to a different distribution of Ir, such as the formation of IrO_x_ nanoclusters and nanoparticles, which cause less structural disruption compared to atomically dispersed Ir [38].

X‐ray photoelectron spectroscopy (XPS) was used to examine the surface chemical state of Ir species in the NiO nanosponge. Figure 4b shows the survey spectrum of samples prepared by sonication in Ir precursor solutions with different concentrations. The main elements detected in all samples are Ni, O, and adventitious carbon (C 1s). Small peaks corresponding to F 1s and P 2p appear due to the anodization electrolyte (NH_4_F and H_2_PO_3_), while a weak Cl 2p peak is observed in NiO‐x Ir, originating from the H_2_IrCl_6_ precursor. A signal from the Ir 4d region is also visible in the NiO‐x Ir electrodes, and its intensity increases with higher Ir concentration, confirming the successful deposition of Ir on the surface.

Figure 4c presents the high‐resolution overlay spectra of the Ir 4f region for all electrodes. As the Ir precursor concentration increases, the Ir 4f peak becomes more intense, suggesting that Ir is increasingly present on the surface, possibly interacting with NiO. The atomic concentrations calculated from the XPS data are shown in Figure 4d and Table S2. The results indicate that the surface Ir content rises with higher precursor concentration. For example, the NiO‐0.5 Ir sample contains 1.6 at% Ir and exhibits the highest Ni signal among all Ir‐decorated electrodes (29.0 at%), while the oxygen atomic percentage decreases, possibly indicating an Ir‐Ni interaction. Moreover, the oxygen and nickel atomic percentages further decrease with increasing Ir loading (1 and 2 mM), suggesting enhanced surface coverage by Ir species and a corresponding reduction in the detectable NiO surface signal. The complete set of atomic concentration values for Ir 4f, Ni 2p, O 1s, and C 1s is provided in Table S2.

The high‐resolution Ni 2p spectra of NiO, NiO‐0.5 Ir, and NiO‐2 Ir in Figure 4e exhibit the expected spin‐orbit doublet, comprising Ni 2p_3/2_ and Ni 2p_1/2_ components. Deconvolution reveals signals attributable to both Ni^2+^ and Ni^3+^ oxidation states. Specifically, the Ni^2+^and Ni^3+^ peaks appear at ∼854.7 and 856.5 eV within the Ni 2p_3/2_ region, and at ∼872.5 and 874.5 eV within the Ni 2p_1/2_ region, respectively. Two satellite peaks are also observed [39]. The calculated Ni^3+^/Ni^2+^ ratio increases from 0.8 in NiO to 0.85 in NiO‐0.5 Ir and further to 1.12 in NiO‐2 Ir, indicating that higher Ir loading promotes Ni oxidation. This behavior is attributed to electronic interactions between Ir species and defect sites in the NiO support, which induce charge redistribution in the NiO lattice and stabilize higher Ni^3+^ states through the formation of Ni─O─Ir coordination environments [38]. The O 1s spectra in Figure S5a for NiO, NiO‐0.5 Ir, and NiO‐2 Ir show peaks at 529.5, 531.2, and 533.0 eV, corresponding to lattice oxygen (Ni─O), surface hydroxyl groups (Ni─OH), and adsorbed water, respectively [40]. With increasing Ir content, the proportion of Ni─O decreases, while the proportion of Ni─OH increases, indicating surface hydroxylation is enhanced with Ir incorporation.

To investigate the effect of sonication on NiO, a control sample (NiO‐sono/0 Ir) was prepared under the same sonication conditions without adding the Ir precursor. High‐resolution Ni 2p spectra of NiO‐sono/0 Ir and NiO in Figure S6a show that the Ni^3+^/Ni^2+^ ratio increases from 0.8 in pristine NiO to 1.0 in NiO‐sono/0 Ir. This observation indicates that sonication induces partial oxidation of Ni^2+^ species. Furthermore, the O 1s spectra (Figure S6b) show decreased lattice oxygen (Ni─O) and increased surface hydroxyl species (Ni─OH), indicating that sonication increases the surface hydroxylation of NiO.

The sonochemical treatment plays an important role in creating defect sites and anchoring Ir species. The formation and collapse of bubbles generate localized high‐energy conditions, which can break bonds and form oxygen vacancies on the NiO surface and induce partial oxidation of Ni^2+^ to Ni^3+^ species in NiO. These defect sites act as favorable sites for Ir anchoring, leading to strong interaction between Ir and the NiO support. As a result, Ir can be stabilized in a highly dispersed form and effectively interacts with the NiO matrix [17, 41].

Figure 4f presents the deconvoluted high‐resolution XPS spectra of the Ir 4f region for NiO‐0.5 Ir and NiO‐2 Ir samples. In the case of NiO‐0.5 Ir, the spectrum can be split into six distinct peaks. The signals observed at 61.5 and 64.5 eV are attributed to the Ir^3+^ 4f_7/2_ and 4f_5/2_ orbitals, respectively, while those at 62.3 and 65.3 eV correspond to the Ir^4+^ oxidation state, confirming the predominant presence of oxidized Ir species on the NiO support [42]. Two additional peaks at 63.5 and 66.5 eV are characteristic of satellite features. In contrast, the NiO‐2 Ir sample exhibits a prominent doublet at 62.3 and 65.3 eV, characteristic of the Ir^4+^ oxidation state and assigned to the 4f_7/2_ and 4f_5/2_ spin‐orbit components. A weaker set of peaks at 63.5 and 66.5 eV also corresponds to satellite features [43]. As shown in Figure S5c, high‐resolution Ir 4d XPS spectra were recorded to further confirm the presence of oxidized Ir species. The Ir 4d spectra show a clear spin‐orbit doublet for both NiO‐0.5 Ir and NiO‐2 Ir electrodes with relatively broad features (FWHM ∼3–4 eV). The peaks at 297.8 and 313.2 eV are assigned to the Ir 4d_5/2_ and Ir 4d_3/2_ orbitals, respectively [44]. The overall dominance of the higher oxidation states suggests that most Ir atoms exist in oxidized forms. These oxidized Ir species are known to exhibit strong interactions with hydroxide ions and OER intermediates, which may enhance their catalytic performance toward the oxygen evolution reaction (OER) [42, 43, 45]. However, higher oxidation states of Ir can lead to the formation of IrO_x_ nanoparticles and the agglomeration of single atoms [43].

To gain further insight into the distribution of Ir species on the NiO surface, ToF‐SIMS analysis combined with principal component analysis (PCA) was performed, with particular focus on verifying the incorporation of Ir single atoms in the NiO‐0.5 Ir sample. The negative ion spectra for pristine NiO, NiO‐0.5 Ir, and NiO‐2 Ir are presented in Figure 5a. According to the characteristic isotope pattern of IrO_2_^−^, two prominent peaks were observed at m/z 222.95 and 224.95 for the IrO_2_^−^ ion, and at m/z 223.96 and 225.96 for the IrO_2_H^−^ ion in both Ir‐containing samples (Figure 5a). A sharp signal at m/z 224.95 in Figure 5b and Figure S7a confirms the presence of IrO_2_^−^ fragments. Notably, the intensity of these IrO_2_^−^ signals is significantly higher in NiO‐2 Ir, indicating a higher concentration of oxidized Ir species, consistent with XPS findings. Additionally, a peak at m/z 73.93, corresponding to the NiO^−^ fragment, appears with comparable intensity in all three samples (Figure S7b). To provide further confirmation of Ir interaction with Ni, the NiIr^−^ fragment was detected at m/z 250.97, cf. Figure 5c, with the highest intensity observed for the NiO‐0.5 Ir electrode. Such substrate‐adsorbate combination fragments are highly indicative of an actual bond formation, indicating that a fraction of the Ir is bonded to Ni in Ir─Ni bonds [7, 46].

**FIGURE 5:**
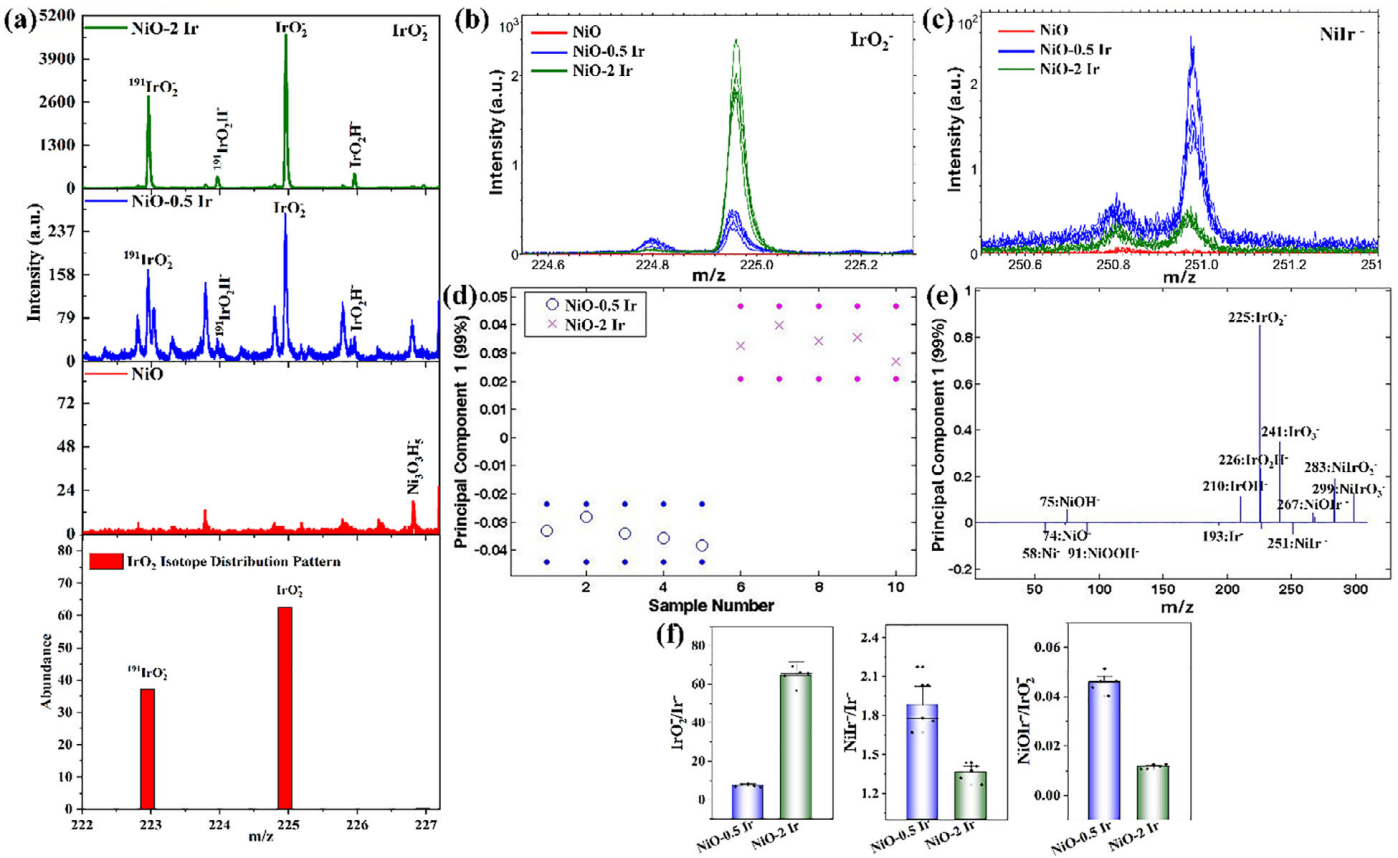
(a) Negative ToF‐SIMS spectra of pristine NiO, NiO‐0.5 Ir, and NiO‐2 Ir electrodes, including the isotope distribution pattern of IrO_2_^−^. (b,c) Negative ToF‐SIMS molecular fragments from five spectra of each pristine NiO, NiO‐0.5 Ir, and NiO‐2 Ir electrodes: (b) IrO_2_^−^ and (c) NiIr^−^; (d,e) PCA of ToF‐SIMS data for NiO‐0.5 Ir and NiO‐2 Ir: (d) Score plot from PCA of the negative ion spectra and (e) corresponding loading plot (signals labeled with their mass and fragment assignment). The dotted lines in the score plot indicate the 95% confidence limits; (f) ToF‐SIMS peak intensity ratios indicate the levels of oxide index, support‐bound index, and support‐vs‐oxide contrast for NiO‐0.5 Ir and NiO‐2 Ir. All ratios were normalized to the total ion intensity, and the error bars represent the standard deviations.

The PCA score plot in Figure 5d shows a clear separation between NiO‐0.5 Ir and NiO‐2 Ir electrodes, with the two sample groups representing the relationships among the spectra positioned on opposite sides of the zero axis. This separation pattern is reflected in the corresponding loading plot in Figure 5e, where fragments with negative or positive loadings are associated with the respective sample groups, highlighting the mass fragments most responsible for the observed distinction (see Table S3 for the list of relevant fragments) [47]. These results suggest that varying the Ir precursor concentration in solution influences how Ir species bind to the surface. Specifically, sonochemical decoration with 0.5 mM Ir leads to a different interaction on the nanostructured NiO substrate than 2 mM Ir, with the former exhibiting a higher abundance of Ni─Ir bond fragments (NiIr^−^) rather than oxide‐related fragments (IrO_2_^−^). To differentiate atomically dispersed Ir species from IrO_2_ nanoclusters and nanoparticles, three diagnostic fragment ratios were evaluated (Figure 5f). The oxide index (IrO_2_^−^/Ir^−^) reflects the relative abundance of oxide‐related fragments, with higher values indicating a greater fraction of IrO_2_ nanoclusters and nanoparticles and lower values consistent with isolated Ir atoms (7.3 ± 0.34 for NiO‐0.5 Ir and 64.4 ± 2.10 for NiO‐2 Ir), in agreement with the XPS results shown in Figure 4f. The Ir‐Ni index (NiIr^−^/Ir^−^) reflects the Ir‐Ni interaction, where higher values indicate anchoring of Ir to the Ni surface, characteristic of single‐atom sites (1.9 ± 0.09 for NiO‐0.5 Ir and 1.36 ± 0.03 for NiO‐2 Ir). The support composite‐oxide contrast (NiOIr^−^/IrO_2_^−^) compares NiO‐bound Ir with oxide fragments, with higher values indicating that Ir is predominantly integrated into the NiO support (0.046 ± 0.002 for NiO‐0.5 Ir and 0.011 ± 0.0004 for NiO‐2 Ir).

### 3.4 Electrocatalytic Performance

The interfacial redox behavior was investigated through steady‐state cyclic voltammetry (Figure 6a). The CV profiles of nanostructured NiO and NiO‐x Ir exhibit distinct redox peaks (oxidation and reduction reactions), indicative of faradaic activity with pseudocapacitive behavior. The asymmetrical shape of the CV curves reflects the quasi‐reversible nature of the redox process represented by Equation (3) [48]:

(3) $$NiO+OH^{-}⇌\mathrm{NiOOH}+e^{-}$$

**FIGURE 6:**
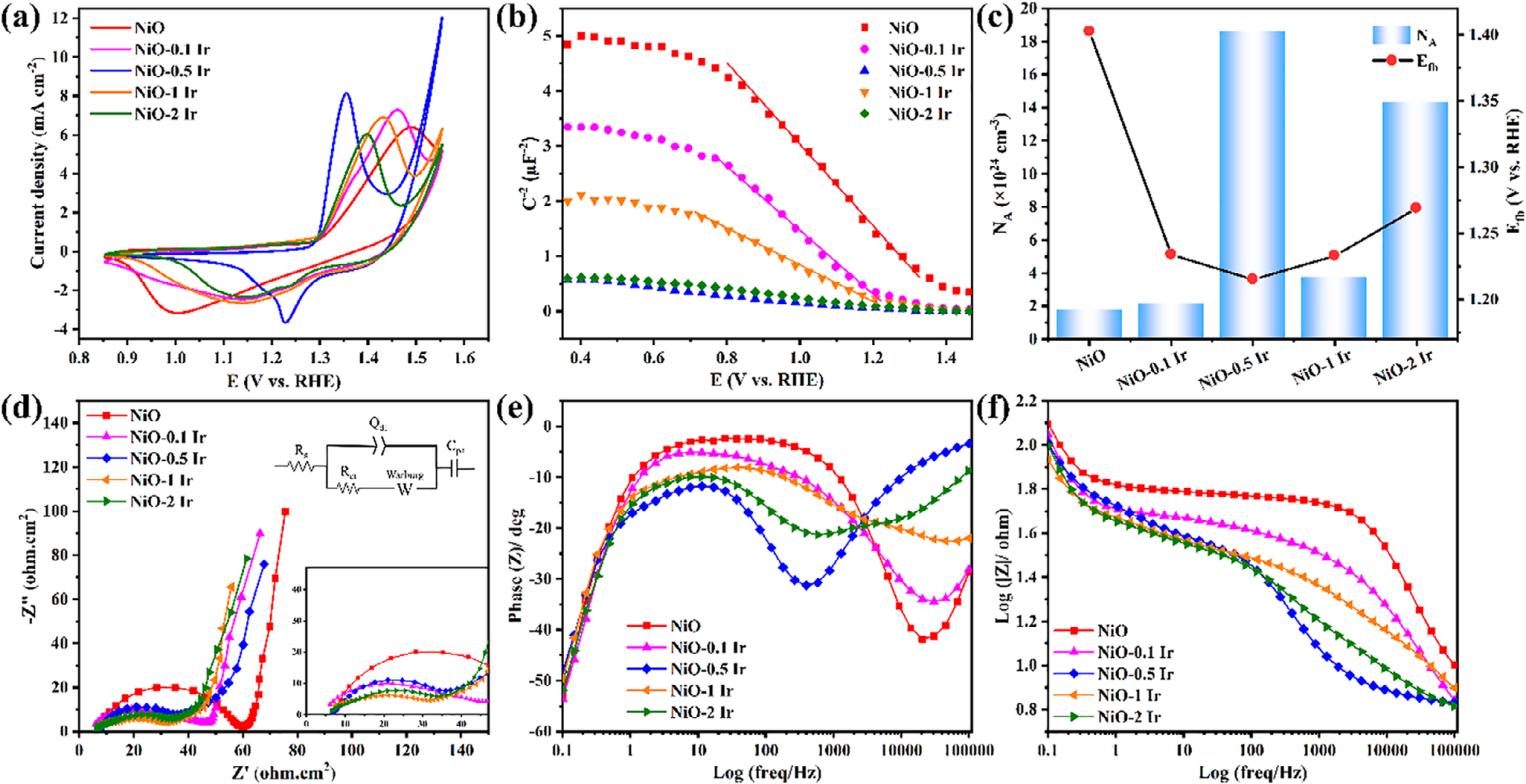
(a) Steady‐state cyclic voltammetry of pristine NiO and NiO‐x Ir at a scan rate of 5 mV s^−1^ in 1.0 M KOH electrolyte; (b) Mott–Schottky plots of pristine NiO and sonochemically decorated NiO with Ir (NiO‐x Ir electrodes), measured at a frequency of 15 kHz in a DC voltage range from −0.5–0.6 V vs. Hg/HgO in 1.0 M KOH; (c) Comparative plots showing changes in charge carrier density and flat band potential extracted from Mott‐Schottky plots with increasing Ir precursor concentration on NiO; (d) Nyquist plots of pristine NiO and NiO‐x Ir, recorded at an AC amplitude of 10 mV in 1.0 M KOH (inset: corresponding equivalent circuit); (e, f) Bode plots of (e) phase angle and (f) total impedance (|Z|) for pristine NiO and Ir‐decorated NiO (NiO‐x Ir) at an AC amplitude of 10 mV in 1.0 M KOH.

This electrochemical transition corresponds to the oxidation of Ni^2+^ to Ni^3+^, involving hydroxide ions from the alkaline electrolyte.

The anodic and cathodic features observed in the voltammograms in Figure 6a correspond to the Ni^2+^/Ni^3+^ redox transitions. These redox reactions involve coupled proton‐electron transfer steps, and their potential shifts with increasing Ir incorporation indicate that Ir surface decoration alters the protonation and deprotonation behavior. The separation between anodic and cathodic peaks (Ni^2+^/Ni^3+^ redox couple) is largest for pristine NiO, indicating that the redox process is electrochemically less reversible due to sluggish interfacial electron transfer. Conversely, this separation is smallest for NiO‐0.5 Ir, indicating enhanced electron transfer kinetics [49].

Figure 6b shows the Mott‐Schottky plots obtained from capacitance values measured by electrochemical impedance spectroscopy, which represent the correlation between the capacitance (C) and the applied potential (V). The linear regions of these plots exhibit a negative slope, confirming the p‐type semiconductor characteristics of the samples [50, 51]. The charge carrier density (N_A_) can be calculated from the slope of the Mott–Schottky curve, as described by Equation (4) [32].

(4) $$\left|slope\right|=\frac{2}{\varepsilon \varepsilon_{0}A^{2}eN_{A}}\;$$

where ɛ_0_ denotes the vacuum permittivity (8.86 × 10^−12^ F m^−1^), ɛ the dielectric constant of the semiconductor (ɛ = 11.9 for NiO_X_), A the contact area, and e the elementary charge [52]. The flat‐band potential (E_fb_) is determined by extrapolating the linear section of the plot to its intersection with the potential axis. The relationship between the space charge layer capacitance (C_sc_) and the semiconductor flat‐band potential (E_fb_) is defined by Equation (5).

(5) $$\frac{1}{C_{sc}^{2}}=-\frac{2}{\varepsilon \varepsilon_{0}A^{2}eN_{A}}\left(E-E_{FB}-\frac{kT}{e}\right)$$

where *k* (1.380649 × 10^−23^ J·K^−1^) is the Boltzmann constant, and *T* is the absolute temperature [32, 50]. The space charge capacitance (C_sc_) at semiconductor/electrolyte interfaces corresponds to charge accumulation [53]. Mott–Schottky analysis is useful only when the semiconductor is under a carrier depletion width. In p‐type semiconductors like NiO, depletion occurs when holes migrate from the semiconductor to the electrolyte, resulting in downward band bending at the interface. This condition is typically achieved by applying a potential more negative than the Fermi level of the semiconductor. To ensure accurate analysis, a broad potential window (−0.5–0.6 V vs. Hg/HgO) was used to observe the transition from C to flat‐band behavior in the Mott–Schottky plots [50].

As shown in Figure 6b,c, the carrier density (i.e., the density of interfacial holes) increases from 1.7 × 10^24^ cm^−3^ in pristine NiO to 2.1 × 10^24^ cm^−3^ in NiO decorated with 0.1 mM Ir (NiO‐0.1 Ir). Since Ir decoration occurred simultaneously with defect formation, variations in Ir precursor concentration influenced the carrier density. With 0.5 mM Ir SACs (NiO‐0.5 Ir), the charge carrier concentration reaches its maximum of 18.6 × 10^24^ cm^−3^. However, further increasing the Ir concentration to 1 mM (NiO‐1 Ir) results in a decrease in carrier density to 3.7 × 10^24^ cm^−3^. Interestingly, for NiO‐2 Ir, the carrier density increases again (14.3 × 10^24^ cm^−3^), which can be attributed to the formation of IrO_x_ nanoclusters or nanoparticles. This suggests that the 1 mM Ir concentration represents a transition phase between atomic dispersion and nanoparticle formation.

The flat band potential (E_fb_) exhibits a decreasing trend following Ir decoration via the sonochemical method, reaching its most negative value at 0.5 mM Ir concentration, then E_fb_ shifts back toward more positive values. This pattern, illustrated by the correlation between carrier density (N_A_) and E_fb_ in Figure 6c (with numerical data in Table S4), reflects a strong interplay between interfacial electronic properties and the Ni^2+^/Ni^3+^ redox couple (Figure 6a). These findings suggest that variations in Ir loading significantly affect the band structure, likely through modulation of charge accumulation and surface chemistry. At potentials above E_fb_, the surface enters a hole accumulation regime, where holes from the electrolyte are retained at the interface either through Ni oxidation or the generation of oxygen ligand holes (Figure 6a) [53, 54]. The optimized electronic structure and surface properties of NiO‐0.5 Ir, reflected in its high density of active sites and low charge accumulation onset, provide a strong basis for its enhanced OER activity, which is explored in detail in Figure 7. These results demonstrate that NiO serves not only as a structural support but also as an electronically active matrix that promotes charge accumulation, defect formation, and strong interaction with Ir species during OER.

**FIGURE 7:**
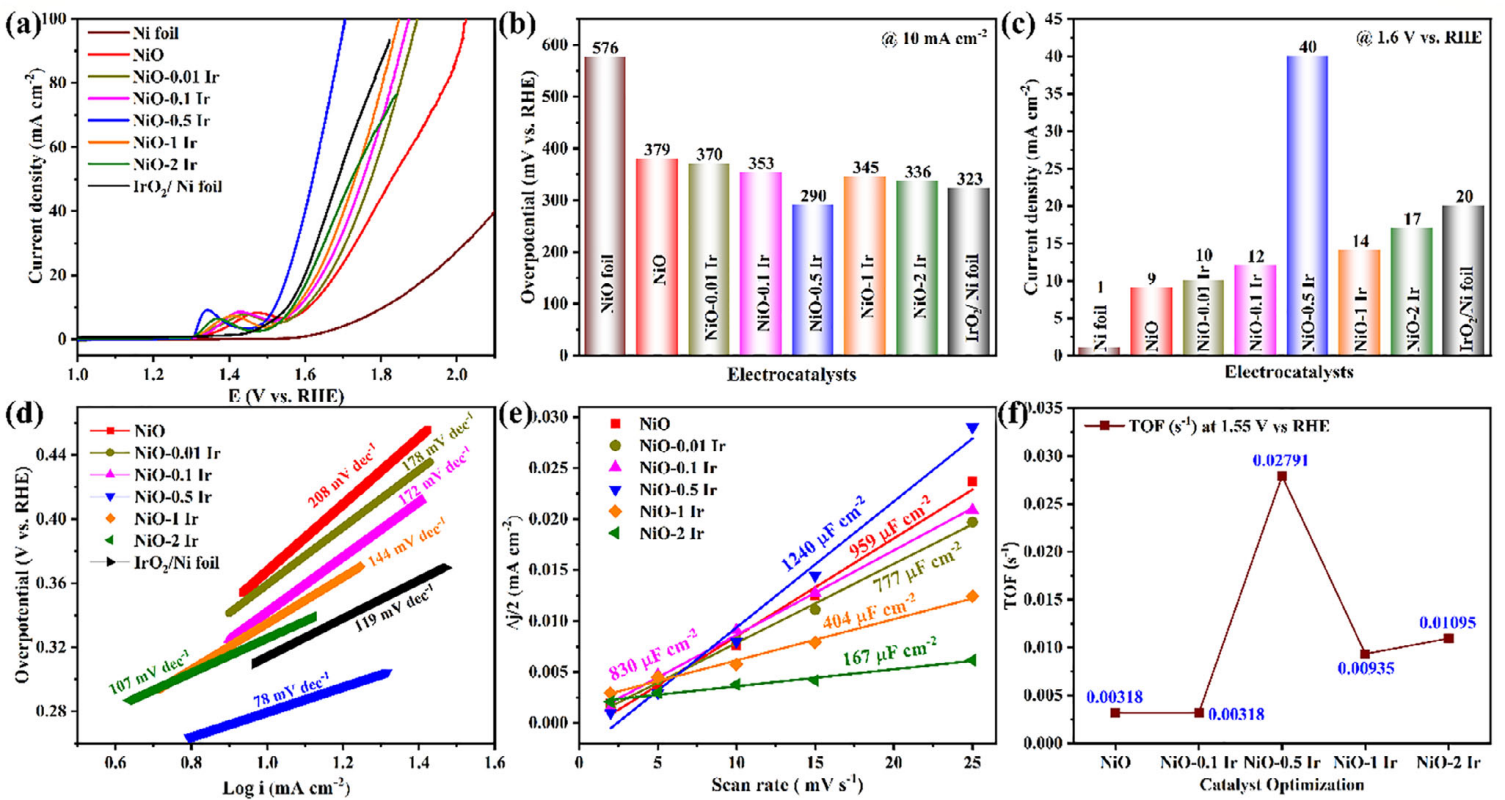
(a‐d) Electrocatalytic performance toward the OER for Ni foil, pristine NiO, sonochemically decorated NiO with Ir (NiO‐x Ir electrodes), and IrO_2_/Ni foil: (a) OER LSV polarization curves, (b) overpotentials required to reach 10 mA cm^−^^2^, (c) current density achieved at 1.6 V vs. RHE, and (d) corresponding Tafel plots in 1.0 M KOH; (e) Calculated double‐layer capacitance (C_dl_) from Δj/2 vs. scan rate plots at 1 V vs. RHE; (f) Comparison of Turnover of frequencies at 1.55 V vs RHE, where the current was retrieved from the backward CV scan.

Electrochemical impedance spectroscopy (EIS) was performed to analyze charge transport dynamics at different Ir loadings. The Nyquist (Figure 6d) and Bode plots, which consist of phase angle and impedance modulus vs. frequency (Figure 6e,f), show the impedance characteristics of the catalysts. In the Nyquist plots (Figure 6d), a small semicircle appears in the high‐frequency region, representing the interfacial charge transfer resistance (R_ct_), and the near‐vertical line at low frequencies reflects pseudocapacitive properties [55]. In the high‐frequency region, the system shows capacitive behavior related to the electric double layer at the electrode‐electrolyte interface, consistent with the CV results shown in Figure 6a. The NiO‐0.5 Ir and NiO‐1 Ir samples show the smallest semicircle radius, corresponding to R_ct_ values of 29.0 and 27.5 Ω, respectively. This is lower than the values recorded for pristine NiO (52.5 Ω) and other variants (Table S4), indicating significantly enhanced charge transfer and more efficient electrochemical kinetics. The corresponding Bode phase diagrams (Figure 6e) showed that in the low‐frequency region, the phase angle deviation increases with the Ir precursor concentration up to 0.5 mM. This demonstrates improved surface oxidation and the formation of adsorbed OH^−^ intermediates [56]. At higher Ir concentrations, the phase deviation decreases again. In the high‐frequency region, the NiO‐0.5 Ir electrode exhibits a phase angle closest to zero, indicating more efficient interfacial charge transfer, in line with the Nyquist data.

The electrocatalytic OER performance of the synthesized catalysts, along with IrO_2_/ Ni foil for comparison, was assessed using a conventional three‐electrode system in 1.0 M KOH. The linear sweep voltammetry (LSV) polarization curves in Figure 7a show that, after anodization to nanostructured NiO, the OER activity improves significantly, reducing the overpotential from 576 to 379 mV at a current density of 10 mA cm^−2^. Upon sonochemical decoration with Ir, NiO‐0.5 Ir exhibits outstanding OER kinetics, requiring an overpotential of only 290 mV to achieve 10 mA cm^−2^ (Figure 7a,b). Notably, this performance surpasses that of IrO_2_/ Ni foil (η_10_ = 323 mV) and outperforms other Ir‐modified samples: NiO‐2 Ir (η_10_ = 336 mV), NiO‐1 Ir (η_10_ = 345 mV), NiO‐0.1 Ir (η_10_ = 353 mV), and NiO‐0.01 Ir (η_10_ = 370 mV) (Table S5). Based on the structural characteristics of the NiO‐x Ir electrodes, NiO‐0.5 Ir features SAC Ir doping, while NiO‐2 Ir contains IrO_x_ nanoclusters or nanoparticles and a higher concentration of surface Ni^3+^ species. This suggests that the improvement in OER performance upon Ir incorporation is due to a synergistic effect: both the presence of surface Ni^3+^ and atomically dispersed Ir contribute to accelerating water oxidation. However, when the Ir loading becomes excessive, as in NiO‐2 Ir, the formation of IrO_x_ nanoclusters and nanoparticles may block active Ni^3+^ sites, thereby limiting further enhancement [38]. All electrocatalysts exhibit a prominent oxidation peak before the onset of OER (Figure 7a), attributed to the Ni^2+^/Ni^3+^ redox transition, which is also evident in the CV curves (Figure 6a). The anodic shift of the Ni^2+^/Ni^3+^ peak to lower potentials in the NiO‐x Ir samples, most notably in NiO‐0.5 Ir, suggests enhanced metal‐support interaction and electronic modification of the NiO matrix due to Ir incorporation [34, 42]. In addition, at 1.6 V vs RHE, NiO‐0.5 Ir delivers the highest current density among all samples (j_1.6V_ = 40 mA cm^−2^), outperforming NiO‐2 Ir, IrO_2_/Ni foil, and pristine NiO by approximately 2.3‐, 2‐, and 4.4‐fold, respectively (Figure 7c). NiO‐0.5 Ir exhibits an impressively low Tafel slope of 78 mV dec^−1^, compared to IrO_2_/Ni foil (119 mV dec^−1^) and pristine NiO (208 mV dec^−1^). It also displays the lowest Tafel slope among all Ir‐modified electrodes, including NiO‐2 Ir (107 mV dec^−1^), NiO‐1 Ir (144 mV dec^−1^), NiO‐0.1 Ir (172 mV dec^−1^), and NiO‐0.01 Ir (178 mV dec^−1^), confirming its outstanding kinetic advantage for the oxygen evolution reaction (OER) (Figure 7d and Table S5).

To establish a direct correlation between carrier density and OER performance, the overpotential at 10 mV cm^−2^ and Tafel slope were plotted as a function of carrier density, which was measured from Mott‐Schottky analysis (Figure S8). An approximate linear inverse correlation is observed (linear fit with R^2^ ≈ 0.87), where increasing carrier density leads to both lower overpotential and smaller Tafel slope. This indicates that enhancing the interfacial hole concentration not only reduces the energetic barrier for OER but also accelerates the reaction kinetics. Among all samples, NiO‐0.5 Ir shows the highest carrier density, with the lowest overpotential and Tafel slope. This confirms that carrier density plays a key role in determining catalytic activity. The improvement can be attributed to the higher availability of holes in p‐type NiO, which enhances interfacial charge transfer and promotes the Ni^2+^/Ni^3+^ redox transition. This process facilitates the formation of catalytically active NiOOH species, thereby accelerating OER kinetics [53, 57].

To clarify the contribution of sonication to defect formation and Ir incorporation, two control samples (NiO‐sono/0 Ir and NiO‐dip/0.5 Ir) were introduced. NiO‐sono/0 Ir refers to sonicated NiO without Ir synthesized under identical conditions as the Ir‐containing samples, while NiO‐dip/0.5 Ir refers to NiO loaded with 0.5 mM Ir via electroless impregnation without sonication. The electrocatalytic OER performance of NiO‐sono/0 Ir and NiO‐dip/0.5 Ir, together with the previously reported samples NiO, NiO‐0.5 Ir, and IrO_2_/Ni foil, are presented in a single comparison (Figure S9) to separate the effects of defects and Ir incorporation.

The electrochemical results (Figure S9) show that NiO‐sono/0 Ir exhibits lower OER activity than pristine NiO, with an overpotential of 478 mV at 10 mA cm^−2^, a Tafel slope of 197 mV dec^−1^, and a current density of 3 mA cm^−2^ at 1.6 V vs. RHE. This indicates that sonication alone does not enhance OER activity and may introduce disorder‐related charge trapping effects in NiO. In contrast, NiO‐dip/0.5 Ir shows improved performance compared to both NiO and NiO‐sono/0 Ir, with an overpotential of 341 mV at 10 mA cm^−2^, a Tafel slope of 168 mV dec^−1^, and a current density of 14 mA cm^−2^ at 1.6 V vs. RHE. This confirms that Ir incorporation enhances the catalytic activity even without sonication‐induced defect formation. However, NiO‐0.5 Ir (sonication + Ir incorporation) shows the best OER performance among all samples. The current density at 1.6 V vs. RHE for NiO‐0.5 Ir is approximately 3‐fold higher than that of NiO‐dip/0.5 Ir. This indicates a clear synergistic effect between sonication‐induced defect sites and Ir incorporation. In this case, the defect‐rich NiO provides more anchoring sites for Ir species, which strengthens metal‐support interaction, improves charge transfer, and stabilizes atomically dispersed Ir active centers.

To gain further insight into the interfacial properties, electrochemically active surface area (ECSA) analysis was conducted. Cyclic voltammetry (CV) was performed at scan rates ranging from 2 to 25 mV s^−1^ at 1 V vs. RHE, as shown in Figure S10. The non‐faradaic region exhibits a symmetric current response with a linear Δj/2 vs. scan rate relationship, where the slope represents the double‐layer capacitance (C_dl_) (Figure 7e). Based on the extracted C_dl_ values, the corresponding ECSA was determined using the specific capacitance (C_s_) (Figure S11) [43, 58]. A constant C_s_ value of NiO was applied to all samples to ensure consistent comparison across the NiO‐x Ir series. Therefore, the ECSA values are approximate and are mainly used for comparing relative trends in electrochemically active surface area. The C_dl_ and ECSA values shown in Figure 7e and Figure S11 indicate that sonication in 0.01 mM Ir leads to a decrease in double‐layer capacitance and, consequently, a reduction in the active surface area. This decrease is attributed to the formation of defects without sufficient decoration of Ir on the defect sites, resulting in active surface areas of 19 cm^2^ for NiO‐0.01 Ir and 21 cm^2^ for NiO‐0.1 Ir. When the Ir concentration reaches 0.5 mM, the maximum active surface area is achieved, corresponding to a 1.3‐fold enhancement from 24 cm^2^ for pristine NiO to 31 cm^2^ for NiO‐0.5 Ir. However, with a further increase in the Ir precursor concentration, the active surface area decreases to 10 cm^2^ and 4 cm^2^ for NiO‐1 Ir and NiO‐2 Ir, respectively (Figure S11).

Turnover frequency (TOF) serves as a robust descriptor of intrinsic catalytic activity, independent of current density, parasitic reactions, and capacitive effects, thereby enabling meaningful comparison and rational design of electrocatalysts for water electrolysis. Its accurate determination, however, relies on reliable quantification of active sites. For metal‐based systems, redox‐derived approaches are particularly effective, especially in the oxygen evolution reaction, where metal centers cycle between oxidation states; for example, the Ni^3+^/Ni^2+^ reduction peak provides a direct measure of electrochemically active surface sites while excluding inactive bulk species [31, 59].

The TOF values (Figure 7f) reveal an increase in TOF reaching an optimal point at NiO‐0.5 Ir and dropping when the Ir is more than 1 mM. An approximately tenfold increase in the TOF for NiO‐0.5 Ir (0.028) relative to NiO (0.003) indicates substantially enhanced intrinsic activity. This improvement is attributed to optimized electronic structure modulation arising from Ni‐Ir coupling, which enhances the catalytic properties of active sites. At lower Ir loading (0.1 mM), the density of atomically dispersed Ir atoms appears insufficient to produce a notable effect, whereas at higher loadings (1 and 2 mM Ir), atomic modulation of the Ni electronic structure is minimal. This interpretation is consistent with FE‐SEM, HR‐TEM, HAADF‐STEM, XPS, and ToF‐SIMS analyses, which suggest the formation of IrO_x_ nanoclusters and nanoparticles at high loadings, in contrast to the atomically dispersed Ir observed in the optimized NiO‐0.5 Ir catalyst.

### 3.5 Electrochemical Stability and Surface Characterization

In addition to the excellent catalytic activity of single‐atom catalysts, their long‐term durability raises concerns under severe oxidative operating conditions [19]. The durability evaluation of catalysts was performed by chronopotentiometry analysis (Figure 8a). The NiO‐0.5 Ir and NiO‐2 Ir show excellent stability for 24 h at 10 and 20 mA cm^−2^ during the alkaline OER, with a low degradation rate. Accelerated cycling tests confirm the durability of the catalysts. As shown in Figure 8b, the polarization curves before and after 1000 CV cycles indicate that both NiO‐0.5 Ir and NiO‐2 Ir electrodes maintain their catalytic activity and stability.

**FIGURE 8:**
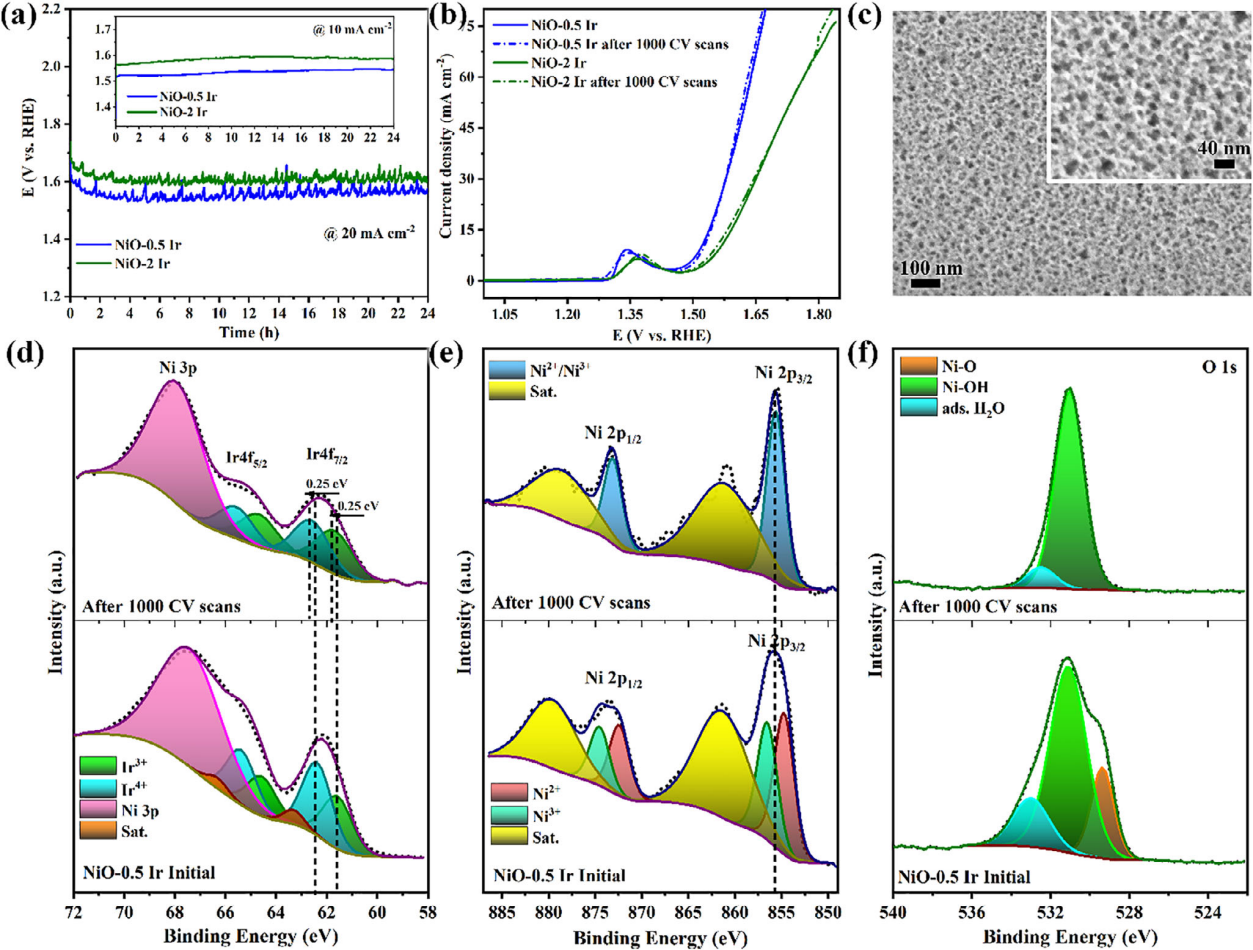
(a) Long‐term stability measurements of NiO‐0.5 Ir and NiO‐2 Ir electrodes at a current density of 20 mA cm^−2^ for 24 h, Inset: stability measurements at 10 mA cm^−2^ for 24 h; (b) Stability evaluation of NiO‐0.5 Ir and NiO‐2 Ir after 1000 CV cycles compared with the initial state; all tests were performed in 1.0 M KOH. (c) FE‐SEM image of the NiO‐0.5 Ir electrode after 1000 CV cycles. XPS spectra of the NiO‐0.5 Ir electrode before and after 1000 CV cycles in 1.0 M KOH at 100 mV s^−1^: (d) deconvolution of Ir 4f spectra; (e) deconvolution of Ni 2p spectra; (f) deconvolution of O 1s spectra.

The stability of the optimal NiO‐0.5 Ir electrode was further assessed by examining its morphology and surface composition after stability testing using FE‐SEM and XPS. FE‐SEM analysis confirms the stability of the porous morphology of NiO‐0.5 Ir after 1000 high‐scan‐rate CV cycles (Figure 8c).

The XPS spectra of NiO‐0.5 Ir in the Ir 4f region (Figure 8d) confirm that Ir remains stable on the surface after 1000 CV cycles. Deconvolution of the Ir 4f peaks shows that both Ir^3+^ and Ir^4+^ shift to higher binding energies by approximately 0.25 eV relative to their positions before the stability test. These changes indicate activation of the active sites and progression to higher oxidation states during the OER. In the Ni 2p region (Figure 8e), the two distinct peaks corresponding to Ni^2+^ and Ni^3+^ merge into a single peak after 1000 CV cycles and show the combined Ni^2+^/Ni^3+^ chemical state at 855.8 eV. This combination is associated with the formation and stabilization of NiOOH or other higher‐valent nickel species [60].

In the O 1s region (Figure 8f), the lattice oxygen peak (Ni‐O) is no longer detectable after 1000 CV cycles, while peaks corresponding to hydroxyl groups (Ni‐OH) and adsorbed H_2_O appear at 531.2 and 533.0 eV, respectively. This shows that more hydroxyl species are present on the electrode surface, indicating the formation or increase of hydroxide‐containing phases [35].

A comparison with representative Ir‐based single‐atom OER catalysts, as summarized in Table S6, shows that NiO‐0.5 Ir achieves competitive performance [6, 13, 19, 34, 36, 38, 61, 62, 63].

## 4 Conclusion

In conclusion, we optimized the anodization time of NiO to achieve a uniform nanostructured surface with significantly enhanced conductivity and the highest oxygen evolution performance. The NiO sample anodized for 3 h exhibited the lowest overpotential of 379 mV at 10 mA cm^−2^, compared to 576 mV for the untreated Ni foil. Then, we successfully anchored single‐atom Ir sites onto the anodically nanostructured NiO using a facile sonochemical method. This approach simultaneously introduces surface defects and decorates the defective NiO with Ir, resulting in a significant enhancement in electrocatalytic activity.

By adjusting the concentration of the Ir precursor, we identified 0.5 mM Ir (NiO‐0.5 Ir) as the optimal loading, which demonstrates the best OER performance. The formation of Ir single atoms (SAs) was confirmed by HR‐TEM, aberration‐corrected HAADF‐STEM, HR‐XPS, and ToF‐SIMS. ToF‐SIMS and PCA show that the NiO‐0.5 Ir electrode exhibits the highest NiIr^−^ fragment intensity and the highest NiIr^−^/Ir^−^ ratio, confirming that a fraction of Ir is bonded to Ni in Ir─Ni bonds characteristic of single‐atom sites. At this optimized concentration, the presence of Ir^3+^ and Ir^4+^ species contributes to an electrochemical oxygen evolution rate nearly 2.3 times higher than that of the NiO‐2 Ir sample, which contains IrO_x_ nanoclusters and nanoparticles. This improvement is attributed to the high density of active Ir sites and the synergistic interaction between high‐valence Ir and Ni species. Mott‐Schottky measurements confirm an 11‐fold increase in carrier density for NiO‐0.5 Ir compared to pristine NiO, along with the lowest flat band potential (1.21 V vs. RHE), indicating a high density of active sites and reduced onset for charge accumulation. The charge transfer resistance decreased to 29.0 Ω, while the electrochemically active surface area (ECSA) increased to 31 cm^2^.

The NiO‐0.5 Ir catalyst showed a low overpotential of 290 mV at 10 mA cm^−2^ and a Tafel slope of 78 mV dec^−1^, representing a 1.5‐fold reduction compared to IrO_2_/ Ni foil and a 2.7‐fold reduction compared to pristine NiO. This strategy provides an effective route for immobilizing single atoms on metal oxide supports without thermal reduction or the use of binders, resulting in a highly active and stable oxygen evolution catalyst with more than 24 h of operational durability and minimal degradation.

## Conflicts of Interest

The authors declare no conflicts of interest.

## Supporting information




**Supporting File**: smll75110‐sup‐0001‐SuppMat.docx.

## Data Availability

The data that support the findings of this study are available from the corresponding author upon reasonable request.
