# Supplementary material for: Ultrasonication‐Induced Iridium Single‐Atom Decoration on Anodized NiO Nanosponge Electrocatalyst for Enhanced Oxygen Evolution Reaction
Source: Small. 2026 Aug 17;22(57):e75110. doi: 10.1002/smll.75110 (PMC13659780; doi:10.1002/smll.75110)
Supplement: Supplementary file 1 [file SMLL-22-e75110-s001.docx]

**Supporting information**

**Ultrasonication-Induced Iridium Single-Atom Decoration on Anodized NiO Nanosponge Electrocatalyst for Enhanced Oxygen Evolution Reaction**

Nastaran Farahbakhsh^1, 2^, Mohammad Hossein Enayati^2,^*, Mahmoud Monirvaghefi^2^, Seyedeh Zahra Sajadian^1^, Jean Marie Vianney Nsanzimana^3^, Majid Shahsanaei^1^, Manuela S. Killian^1,^*

*^1^ Chemistry and Structure of novel Materials, Department of Chemistry and Biology, University of Siegen, Paul-Bonatz-Str. 9-11, 57076 Siegen, Germany*

*^2^ Department of Materials Engineering, Isfahan University of Technology, Isfahan 84156-83111, Iran*

*^3^ Department of Industrial Engineering, University of Padova, Via Marzolo 9, 35131 Padova, Italy*

*^*^ Corresponding author. Email:* [*manuela.killian@uni-siegen.de*](mailto:manuela.killian@uni-siegen.de)*,* [*ena78@iut.ac.ir*](mailto:ena78@iut.ac.ir)

Figure S1. FE-SEM images of (a) 1h NiO, (b) 3h NiO (i.e. NiO), (c) 6h NiO, and (c) 16h NiO (Insets show ion-milled cross-sectional images).

Figure S2. (a-c) Electrocatalytic performance toward the OER for Ni foil, 1 h NiO, 3 h NiO (NiO), 6 h NiO, 16 h NiO, and IrO₂/Ni foil: (a) OER LSV polarization curves, (b) overpotentials required to reach 10 mA cm⁻², and (c) corresponding Tafel plots in 1.0 M KOH; (d) Nyquist plots of 1 h NiO, 3 h NiO (NiO), 6 h NiO, and 16 h NiO, recorded at an AC amplitude of 10 mV in 1.0 M KOH; (e, f) Bode plots of (e) phase angle and (f) total impedance (|Z|) for NiO synthesized with different anodization times, measured at an AC amplitude of 10 mV in 1.0 M KOH.

Table S1. Electrochemical OER performance parameters derived from polarization curves and EIS in 1.0 M KOH for Ni foil, NiO synthesized at different anodization times, and IrO_2_/Ni foil electrodes

| Samples | ɳ_10_  (mV) | b  (mV dec^-1^) | R_ct_  (ohm) |
| --- | --- | --- | --- |
| Ni foil | 576 | 260 | - |
| 1h NiO | 503 | 225 | 464.7 |
| 3h NiO (NiO) | 379 | 208 | 52.5 |
| 6h NiO | 512 | 231 | 518.5 |
| 16h NiO | 556 | 265 | 1370.2 |
| IrO_2_/Ni foil | 323 | 119 | - |

^*^ɳ_10_: Overpotential at 10 mA cm^-2^

^*^b: Tafel slope

^*^R_ct_: Charge transfer resistance

Note S1. The electrocatalytic OER performance of the synthesized nanostructured NiO at different anodization times was investigated using a conventional three-electrode system in 1.0 M KOH (Figure S2, Table S1). The linear sweep voltammetry (LSV) polarization curves in Figure S2a show that after 1 h of anodization, the OER activity improved, reducing the overpotential from 576 mV cm^-2^ for Ni foil to 503 mV cm^-2^ for 1h NiO at a current density of 10 mA cm^-2^. After 3 h of anodization, the performance peaked, with an overpotential of 379 mV cm^-2^ at the same current density. Figure S2c further supports this result, showing the lowest Tafel slope of 208 mV dec^-1^ for the 3h NiO sample. However, increasing the anodization time to 6h and 16h led to a decline in OER performance, with overpotentials rising to 512 mV cm^-2^ and 556 mV cm^-2^, respectively. Moreover, the Nyquist (Figure S2d) and Bode plots (Figures S2e and S2f) illustrate the interfacial characteristics of the catalysts. In the Nyquist plots, a semicircle appears in the high-frequency region, representing the interfacial charge transfer resistance (R_ct_). For the 3h NiO sample, the semicircle is significantly smaller, indicating a reduced R_ct_ of 52.5 Ω.

Figure S3. (a) TEM images of FIB-cut nanostructured NiO; (b) porous structure at the cracked edges of pristine NiO; (c-e) FE-SEM images of NiO-0.01 Ir (c), NiO-0.1 Ir (d), and NiO-1 Ir (e) (insets show higher magnification).

Figure S4. (a,b) Aberration-corrected HAADF-STEM images of NiO-0.5 Ir from different regions, with yellow circles indicating Ir single atoms; (c,d) aberration-corrected HAADF-STEM images of NiO-2 Ir from different regions, with yellow circles marking Ir single atoms and red circles indicating IrO_x_ nanoclusters.

Figure S5. Deconvoluted high-resolution XPS spectra of (a) O 1s and (b) C 1s for NiO, NiO-0.5 Ir, and NiO-2 Ir, and High-resolution XPS spectra of (c) Ir 4d for NiO-0.5 Ir and NiO-2 Ir

Table S2. Atomic surface concentrations of C, O, Ni, and Ir on pristine NiO and Ir-decorated NiO (NiO-x Ir) electrodes, derived from the corresponding high-resolution XPS core-level spectra (C 1s, O 1s, Ni 2p, and Ir 4f)

| Samples | Concentration (at%) | | | |
| --- | --- | --- | --- | --- |
|  | C 1s | O 1s | Ni 2p | Ir 4f |
| NiO | 20.6 | 48.1 | 31.3 | 0 |
| NiO-0.01 Ir | 22.5 | 50.6 | 26.5 | 0.4 |
| NiO-0.1 Ir | 21.9 | 48.6 | 28.6 | 0.9 |
| NiO-0.5 Ir | 22.1 | 47.3 | 29.0 | 1.6 |
| NiO-1 Ir | 26.8 | 46.8 | 23.4 | 3.0 |
| NiO-2 Ir | 27.6 | 47.7 | 18.8 | 5.9 |

Figure S6. Deconvoluted high-resolution XPS spectra of (a) Ni 2p and (b) O 1s for NiO and NiO-sono/0 Ir.

Figure S7. Negative ToF-SIMS molecular fragments of (a) IrO_2_^-^, and (b) NiO^-^ for pristine NiO, NiO-0.5 Ir, and NiO-2 Ir electrodes.

Table S3. Peak list used for PCA analysis of negative-polarity spectra of NiO-0.5Ir and NiO-2Ir. Fragment assignments for each sample were determined based on the loading plot shown in Figure 4e

| m/z | Ion |
| --- | --- |
| 58 | Ni^-^ |
| 74 | NiO^-^ |
| 75 | NiOH^-^ |
| 91 | NiOOH^-^ |
| 193 | Ir^-^ |
| 210 | IrOH^-^ |
| 225 | IrO_2_^-^ |
| 226 | IrO_2_H^-^ |
| 241 | IrO_3_^-^ |
| 251 | NiIr^-^ |
| 267 | NiOIr^-^ |
| 283 | NiIrO_2_^-^ |
| 299 | NiIrO_3_^-^ |

Table S4. Mott-Schottky-derived parameters and EIS results in 1.0 M KOH for pristine NiO and Ir-decorated NiO (NiO-x Ir) electrodes

| Samples | N_A_^*^  (cm^-3^) | E_fb_^*^  (V vs RHE) | R_ct_^*^  (ohm) |
| --- | --- | --- | --- |
| NiO | 1.7×10^24^ | 1.40 | 52.5 |
| NiO-0.1 Ir | 2.1×10^24^ | 1.23 | 42.4 |
| NiO-0.5 Ir | 18.6×10^24^ | 1.21 | 29.0 |
| NiO-1 Ir | 3.7×10^24^ | 1.23 | 27.5 |
| NiO-2 Ir | 14.3×10^24^ | 1.27 | 31.0 |

^*^N_A_: Charge carrier density

^*^E_fb_: Flat-band potential

^*^R_ct_: Charge transfer resistance

Table S5. Electrochemical OER performance parameters derived from polarization curves in 1.0 M KOH for Ni foil, pristine NiO, Ir-decorated NiO (NiO-x Ir), and IrO_2_/Ni foil electrodes

| Samples | ɳ_10_^*^  (mV) | b^*^  (mV dec^-1^) | j_1.6V_^*^  (mA) |
| --- | --- | --- | --- |
| Ni foil | 576 | 260 | 1 |
| NiO | 379 | 208 | 9 |
| NiO-0.01 Ir | 370 | 178 | 10 |
| NiO-0.1 Ir | 353 | 172 | 12 |
| NiO-0.5 Ir | 290 | 78 | 40 |
| NiO-1 Ir | 345 | 144 | 14 |
| NiO-2 Ir | 336 | 107 | 17 |
| IrO_2_/Ni foil | 323 | 119 | 20 |

^*^ɳ_10_: Overpotential at 10 mA cm^-2^

^*^b: Tafel slope

^*^j_1.6V_: Current density at 1.6 V vs. RHE

Figure S8. Correlation between carrier density (N_A_) and OER activity (overpotential, ɳ_10_, and Tafel slope, b), with an approximate linear inverse correlation relationship (R^2^ ≈ 0.87).

Figure S9. Electrocatalytic performance toward the OER for pristine NiO, NiO-0.5 Ir, IrO_2_/Ni foil, NiO-sono/0 Ir, and NiO-dip/0.5 Ir: (a) OER LSV polarization curves, (b) corresponding Tafel plots in 1.0 M KOH, and (c) overpotentials required to reach 10 mA cm⁻² and current density achieved at 1.6 V vs. RHE.

Figure S10. Cyclic voltammograms at non-faradic potential windows with different scan rates: (a) NiO, (b) NiO-0.01 Ir, (c) NiO-0.1 Ir, (d) NiO-0.5 Ir, (e) NiO-1 Ir, and (f) NiO-2 Ir.

Figure S11. Electrochemically active surface area (ECSA) of pristine NiO and NiO-x Ir.

Table S6. Comparison of the OER performance of the NiO-0.5 Ir catalyst with recently reported Ir-based single-atom

| Electrode | Electrolyte | Overpotential | Tafel slope | Current density | Stability | Ref. |
| --- | --- | --- | --- | --- | --- | --- |
| IrO_2_@NiO/NF | 1.0 M KOH | 278 mV at 10 mA cm^-2^ | 41.8 mV dec^-1^ | 98.33 µA cm^-2^ at 1.53 V | 25 h at 10 mA cm^-2^ | [1] |
| Ir_1_/NFS (IrSA/NiFeS) | 1.0 M KOH | 170 mV at 10 mA cm^-2^ | 36 mV dec^-1^ | - | 350 h at 100 mA cm^-2^ | [2] |
| Ir _18 wt%_-NiO | 1.0 M KOH | 215 mV at 10 mA cm^-2^ | 38 mV dec^-1^ | 114 mA cm^-2^ at 260 mV | 10 h at 10 mA cm^-2^ | [3] |
| Ir_1_@Co/NC | 1.0 M KOH | 260 mV at 10 mA cm^-2^ | 163 mV dec^-1^ | - | - | [4] |
| Ir@IrNiO | 1.0 M KOH | 195 mV at 10 mA cm^-2^ | 41 mV dec^-1^ | - | 10 h at 10 mA cm^-2^ | [5] |
| (RuIr)O_2_/C | 0.1 M HClO_4_ | 174 mV at 10 mA cm^-2^ | 41.7 mV dec^-1^ | 4.96 A cm^-2^ at 2.0 V | 360 h at 100 mA cm^-2^ | [6] |
| Ir-doped CoMn_2_O_4_ | 0.05 M H_2_SO_4_ | 232 mV at 10 mA cm^-2^ | 54.5 mV dec^-1^ | - | 1000 h at 250 mA cm^-2^ | [7] |
| Mn-doped RuIr nanocactus | 0.1 M HClO_4_ | 247 mV at 100 mA cm^-2^ | 45 mV dec^-1^ | - | 180 h at 10 mA cm^-2^ | [8] |
| Ir@Sr-p-TiO_2_ NWs | 0.5 M H_2_SO_4_ | 250 mV at 10 mA cm^-2^ | 51.6 mV dec^-1^ | - | 80 h | [9] |
| Ir-doped FeNiP (Ir_25_-Fe_16_Ni_100_P_64_) | 1.0 M KOH | 232 mV at 10 mA cm^-2^ | 48 mV dec^-1^ | - | 10 h at 10 mA cm^-2^ | [10] |
| Ir_SA_-Ni_2_P | 1.0 M KOH | 149 mV at 10 mA cm^-2^ | 90.1 mV dec^-1^ | 154 mA cm^-2^ at 300 mV | 40 h at 10 mA cm^-2^ | [11] |
| Ir_emb_-NiO | 1.0 M KOH | 256 mV at 10 mA cm^-2^ | 41 mV dec^-1^ | 112 mA cm^-2^ at 1.55 V | 20 h at 20 mA cm^-2^ | [12] |
| Ir_SA_-NiFe LDH/NiMo | 1.0 M KOH | 236 mV at 500 mA cm^-2^ | 39 mV dec^-1^ | - | 500 h at 500 mA cm^-2^ | [13] |
| Ir/NiFe LDH | 1.0 M KOH | 202 mV at 10 mA cm^-2^ | 56.1 mV dec^-1^ | - | 200 h at 100 mA cm^-2^ | [14] |
| Ir_SA_-V_O_-CoNiO_2_ | 1.0 M KOH | 183 mV at 10 mA cm^-2^ | 64.8 mV dec^-1^ | - | 25 h at 10/100 mA cm^-2^ | [15] |
| Ir_0.33_@Co_3_O_4_ | 1.0 M KOH | 296 mV at 10 mA cm^-2^ | 68 mV dec^-1^ | - | 2h at 2 to 10 mA cm^-2^ | [16] |
| NiFeIr_0.03_/Ni NW@NSs | 1.0 M KOH | 200 mV at 10 mA cm^-2^ | 44.6 mV dec^-1^ | - | 12 h at 10 mA cm^-2^ | [17] |
| NiO-0.5 Ir | 1.0 M KOH | 290 mV at 10 mA cm^-2^ | 78 mV dec^-1^ | 40 mA cm^-2^ at 1.6 V | 24 h at 10 mA cm^-2^ | This work |

**References**

[1]	J. Liu, Z. Wang, K. Su, D. Xv, D. Zhao, J. Li, H. Tong, D. Qian, C. Yang, Z. Lu, Self-supported hierarchical IrO_2_@ NiO nanoflake arrays as an efficient and durable catalyst for electrochemical oxygen evolution, ACS Appl. Mater. Interfaces. 11 (2019) 25854–25862.

[2]	Z. Lei, W. Cai, Y. Rao, K. Wang, Y. Jiang, Y. Liu, X. Jin, J. Li, Z. Lv, S. Jiao, W. Zhang, P. Yan, S. Zhang, R. Cao, Coordination modulation of iridium single-atom catalyst maximizing water oxidation activity, Nat. Commun. 13 (2022) 1–10. https://doi.org/10.1038/s41467-021-27664-z.

[3]	Q. Wang, X. Huang, Z.L. Zhao, M. Wang, B. Xiang, J. Li, Z. Feng, H. Xu, M. Gu, Ultrahigh-loading of Ir single atoms on NiO matrix to dramatically enhance oxygen evolution reaction, J. Am. Chem. Soc. 142 (2020) 7425–7433.

[4]	W. Lai, L. Zhang, W. Hua, S. Indris, Z. Yan, Z. Hu, B. Zhang, Y. Liu, L. Wang, M. Liu, General π‐electron‐assisted strategy for Ir, Pt, Ru, Pd, Fe, Ni single‐atom electrocatalysts with bifunctional active sites for highly efficient water splitting, Angew. Chemie Int. Ed. 58 (2019) 11868–11873.

[5]	M. Liu, Y. Ji, Y. Li, P. An, J. Zhang, J. Yan, S. Liu, Single‐Atom Doping and High‐Valence State for Synergistic Enhancement of NiO Electrocatalytic Water Oxidation, Small. 17 (2021) 2102448.

[6]	Y. Park, H.Y. Jang, T.K. Lee, T. Kim, D. Kim, D. Kim, H. Baik, J. Choi, T. Kwon, S.J. Yoo, Atomic-level Ru-Ir mixing in rutile-type (RuIr) O_2_ for efficient and durable oxygen evolution catalysis, Nat. Commun. 16 (2025) 579.

[7]	W. Ko, J. Shim, H. Ahn, H.J. Kwon, K. Lee, Y. Jung, W.H. Antink, C.W. Lee, S. Heo, S. Lee, Controlled structural activation of iridium single atom catalyst for high-performance proton exchange membrane water electrolysis, J. Am. Chem. Soc. 147 (2025) 2369–2379.

[8]	J. Joo, Y. Park, J. Kim, T. Kwon, M. Jun, D. Ahn, H. Baik, J.H. Jang, J.Y. Kim, K. Lee, Mn‐Dopant differentiating the Ru and Ir oxidation states in catalytic oxides toward durable oxygen evolution reaction in acidic electrolyte, Small Methods. 6 (2022) 2101236.

[9]	H. Zhu, Y. Wang, Z. Jiang, B. Deng, Y. Xin, Z. Jiang, Defect engineering promoted ultrafine Ir nanoparticle growth and Sr single‐atom adsorption on TiO_2_ nanowires to achieve high‐performance overall water splitting in acidic media, Adv. Energy Mater. 14 (2024) 2303987.

[10]	N. Yang, S. Tian, Y. Feng, Z. Hu, H. Liu, X. Tian, L. Xu, C. Hu, J. Yang, Introducing High‐Valence Iridium Single Atoms into Bimetal Phosphides toward High‐Efficiency Oxygen Evolution and Overall Water Splitting, Small. 19 (2023) 2207253.

[11]	Q. Wang, Z. Zhang, C. Cai, M. Wang, Z.L. Zhao, M. Li, X. Huang, S. Han, H. Zhou, Z. Feng, Single iridium atom doped Ni_2_P catalyst for optimal oxygen evolution, J. Am. Chem. Soc. 143 (2021) 13605–13615.

[12]	A. Kumar, M. Gil-Sepulcre, J.P. Fandré, O. Rüdiger, M.G. Kim, S. DeBeer, H. Tüysüz, Regulating Local Coordination Sphere of Ir Single Atoms at the Atomic Interface for Efficient Oxygen Evolution Reaction, J. Am. Chem. Soc. 146 (2024) 32953–32964.

[13]	Y. Wu, M. Chen, H. Sun, T. Zhou, X. Chen, G. Na, G. Qiu, D. Li, N. Yang, H. Zheng, Coupling Ir single atom with NiFe LDH/NiMo heterointerface toward efficient and durable water splitting at large current density, Appl. Catal. B Environ. Energy. 360 (2025) 124548.

[14]	D. Li, L. Ding, S. Zhang, P. Zhang, P. Claudia, D. Wang, Q. Zhao, In-situ electrostatic anchoring of highly oxidized Ir single atoms on NiFe LDH for efficient water oxidation, Chem. Eng. J. (2025) 165662.

[15]	M.-Q. Yang, K.-L. Zhou, C. Wang, M.-C. Zhang, C.-H. Wang, X. Ke, G. Chen, H. Wang, R.-Z. Wang, Iridium single-atom catalyst coupled with lattice oxygen activated CoNiO_2_ for accelerating the oxygen evolution reaction, J. Mater. Chem. A. 10 (2022) 25692–25700.

[16]	H. Chen, S. Chen, Z. Zhang, L. Sheng, J. Zhao, W. Fu, S. Xi, R. Si, L. Wang, M. Fan, Single-atom-induced adsorption optimization of adjacent sites boosted oxygen evolution reaction, Acs Catal. 12 (2022) 13482–13491.

[17]	X. Luo, X. Wei, H. Zhong, H. Wang, Y. Wu, Q. Wang, W. Gu, M. Gu, S.P. Beckman, C. Zhu, Single-atom Ir-anchored 3D amorphous NiFe nanowire@ nanosheets for boosted oxygen evolution reaction, ACS Appl. Mater. Interfaces. 12 (2019) 3539–3546.
